# Chronic Histological Outcomes of Indirect Traumatic Optic Neuropathy in Adolescent Mice: Persistent Degeneration and Temporally Regulated Glial Responses

**DOI:** 10.3390/cells10123343

**Published:** 2021-11-28

**Authors:** Shelby M. Hetzer, Emily M. Shalosky, Jordyn N. Torrens, Nathan K. Evanson

**Affiliations:** 1 Neuroscience Graduate Program, University of Cincinnati College of Medicine, Cincinnati, OH 45267, USA; canslesy@mail.uc.edu; 2 Department of Biological Sciences, University of Cincinnati, Cincinnati, OH 45221, USA; shalosem@mail.uc.edu; 3 Division of Pediatric Rehabilitation Medicine, Cincinnati Children’s Hospital Medical Center, Cincinnati, OH 45229, USA; torrenjn@mail.uc.edu; 4 Department of Pediatrics, University of Cincinnati, Cincinnati, OH 45229, USA

**Keywords:** traumatic optic neuropathy, head trauma, adolescent head trauma, mice, chronic, gliosis, neurodegeneration

## Abstract

Injury to the optic nerve, termed, traumatic optic neuropathy (TON) is a known comorbidity of traumatic brain injury (TBI) and is now known to cause chronic and progressive retinal thinning up to 35 years after injury. Although animal models of TBI have described the presence of optic nerve degeneration and research exploring acute mechanisms is underway, few studies in humans or animals have examined chronic TON pathophysiology outside the retina. We used a closed-head weight-drop model of TBI/TON in 6-week-old male C57BL/6 mice. Mice were euthanized 7-, 14-, 30-, 90-, and 150-days post-injury (DPI) to assess histological changes in the visual system of the brain spanning a total of 12 regions. We show chronic elevation of FluoroJade-C, indicative of neurodegeneration, throughout the time course. Intriguingly, FJ-C staining revealed a bimodal distribution of mice indicating the possibility of subpopulations that may be more or less susceptible to injury outcomes. Additionally, we show that microglia and astrocytes react to optic nerve damage in both temporally and regionally different ways. Despite these differences, astrogliosis and microglial changes were alleviated between 14–30 DPI in all regions examined, perhaps indicating a potentially critical period for intervention/recovery that may determine chronic outcomes.

## 1. Introduction

An estimated 43% of traumatic brain injury (TBI) survivors will develop chronic impairment (e.g., migraine, motor impairment, memory decline, visual deficits, etc.), amounting to roughly three million long-term disability cases in the US [1,2,3]. Roughly 812,000 of these cases occur in children and adolescents under 17 each year (CDC), making TBI the leading cause of death and disability among this age group [4]. Thus, it is necessary to understand how chronic symptoms develop, how long they take to develop, and how long they may persist, to inform the appropriate treatment of traumatic brain injury in adolescents. A major injury mechanism in TBI is traumatic axonal injury (TAI), which results from shearing acceleration/deceleration forces of impact or compaction of white matter tracts. Chronic white matter damage and gliosis are also major drivers of disability after TBI [5], and TAI is associated with worse psychological and behavioral outcomes in adolescents [6].

Among animal models for TAI, injury to the optic nerve/tract has been reported (e.g., [7,8,9,10]), making it an attractive approach for the study of optic nerve injury due to its well-characterized anatomy. Examination of optic nerve injury through a TAI model is also important because visual impairment occurs in roughly 20% of children with TBI [11]. We previously described an adolescent murine closed-head trauma model characterized by axonal degeneration and gliosis in the optic nerve and its projection targets, retinal ganglion cell death, and behavioral visual impairment [12,13,14]. This injury model appears to produce damage to the optic nerve via force transmission through the optic canal and reproduces key features of indirect traumatic optic neuropathy (iTON). iTON is a chronic TBI-associated problem, and there is evidence of increased retinal deterioration and worsening of visual impairments for at least 35 years after TBI [15]. These findings in optic nerve injury patients parallel rat experimental TBI, in which there is persistent white matter thinning at least six months after injury. Diffusion-weighted/tensor imaging of TAI patients also reveals a long-term decline in white matter pathology at least 8 months after injury [16].

This chronic worsening of axonal pathology is likely due to Wallerian Degeneration (WD), an active degenerative process that is characteristically slower in the central nervous system than the peripheral nervous system (i.e., days vs. months to years) likely due to glial cell activity [17]. WD mechanisms have been well described and reviewed with the general pattern of degeneration proceeding as follows: (1) axon-specific injury, (2) distal axon degeneration, mitochondrial dysfunction, and calcium release, and (3) microglial/oligodendrocyte response (e.g., [18]; reviewed in [17,19,20]). Though WD in the CNS can occur for days to years following axonal injury, follow-up in many studies ends well before the potential onset of chronic pathology.

Interestingly, diffusion tensor imaging studies show the potential for recovery of white matter tracts 30 days after TBI [19]. This potential for recovery is supported through axonal reorganization/recovery in the optic system in rodent models of TAI, although it arises a bit earlier (2–14 days after optic nerve injury) [20,21,22]. Differences in the time course of TAI/WD progression likely vary depending on injury methodologies, the lengths of axons injured, and the presence of cell death. For example, TAI could result in initial cell death and axonal dieback followed by (a) brief recovery of axonal integrity and subsequent decline [23] or (b) continued improvement [18]. Alternatively, TAI (including cases of iTON) is associated with delayed and progressive worsening of axonal injury due to processes of secondary WD via recruitment of glial cells and activation of immune responses in the presence of axonal debris [24]. In support of this, iTON patients showed a delayed presentation of symptoms up to 7–9 years after injury [25], which could indicate unknown early protective or reparative mechanisms of the optic nerve that are not sustainable after TAI particularly in cases where cell death occurs (i.e., retinal cell death).

Thus, examining the long-term histological effects and cellular responses of degeneration following optic nerve injury could improve understanding of diagnosis and treatment of iTON and/or TAI following brain injury. Accordingly, we undertook a long-term follow-up study of the brain histologic response to optic nerve injury after blunt head trauma, in primary and secondary targets of optic nerve projections in adolescent mice. We hypothesized that there would be chronic degeneration of the visual system following head trauma that would follow a pattern of axonal Wallerian degeneration and persist long after the initial insult.

## 2. Materials and Methods

### 2.1. Animals

Experiments were performed in 6-week old adolescent male C57BL/6J mice (Jackson Laboratories, Bar Harbor, ME, USA). Mice were housed under a 14 h:10 h light:dark schedule in pressurized individually ventilated cage racks, with 4 mice per cage, and were given ad libitum access to water and standard rodent chow. Animals habituated to the vivarium for one week prior to undergoing traumatic brain injury and subsequent procedures. The University of Cincinnati Institutional Animal Care and Use Committee approved all experimental procedures (protocol #20-02-26-02, approved 16 April 2020).

### 2.2. Traumatic Brain Injury

The closed-head injury was performed by weight drop, as previously described [12,26]. Briefly, mice were anesthetized using isoflurane (2–3%) until no toe-pinch reflex was present and placed in a prone position under a metal rod (1.2 cm diameter; 400 g) that was raised to 1.5 cm above the intact, unshaven scalp. The head was rested on a 0.5 cm thick piece of corkboard to prevent head displacement and reduce acceleration/deceleration of the weight force. The injury was produced by dropping the rod over the approximate location of bregma. After head trauma, mice were placed under an oxygen hood made from a pipet box connected to an oxygen tank and were given 100% oxygen (O_2_) until normal breathing returned—approximately 1–5 min. Mice were then removed from the chamber and, upon regaining righting reflex, were returned to their home cages. Injured mice will hereon be referred to as TBI mice.

**Figure 1:**
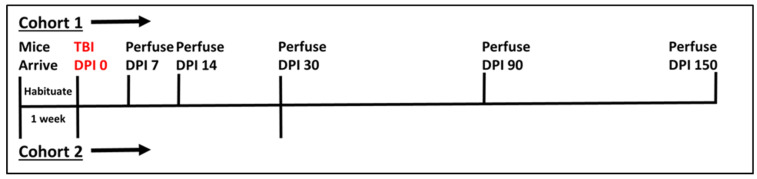
Timeline of experimental procedures. Cohort 1 was our initial experimental cohort with all mice experiencing TBI on the same Day (DPI 0), and subgroups perfused at each timepoint noted. Due to the loss of some tissue samples from cohort 1, however, a second cohort (Cohort 2) was added to the 30 DPI time point. Vertical Lines above the horizontal indicate Cohort 1 while those below the horizontal line are associated with Cohort 2.

Sham animals were anesthetized, weighed, and allowed to recover. Initially, 84 mice underwent the aforementioned procedure and were divided in cohorts of sham and TBI for five chronic time points—7 (sham n = 8, TBI n = 8), 14 (sham n = 8, TBI n = 8), 30 (sham n = 8, TBI n = 8), 90 (sham n = 8, TBI n = 8), and 150 (sham n = 10, TBI n = 10) days post-injury (DPI). The final time point has a higher number of mice because extra mice were ordered to account for expected mortality and those mice that exceeded the power analysis requirement of 8/group were added to this final group. Unfortunately, after initial procedures, four of the eight TBI mice for the 30 DPI time-point were lost, so a new set of eight sham and eight TBI mice were run separately following identical procedures and added to the remaining cohort. See Figure 1 for a timeline for each cohort.

### 2.3. Histology

#### Tissue Collection

For immunohistochemical (IHC) analysis, mice were euthanized using Fatal Plus^®^ 7, 14, 30, 90, and 150 DPI. Mice were perfused transcardially with 4% paraformaldehyde in 0.02 M phosphate-buffered saline (PBS) solution (pH 7.4). Brains were removed and post-fixed in 4% paraformaldehyde overnight in 0.01 M PBS, rinsed in 0.01 M PBS, and immersed in 30% sucrose solution at 4 °C until sectioning (up to 4 months depending on the time point). Sucrose-saturated brains were frozen on dry ice and sectioned at 35 μm using a sliding microtome (Leica, Bannockburn, IL, USA). Sections were stored in cryoprotectant solution (0.01 M PBS, polyvinyl-pyrrolidone (Sigma –Cat# PVP-40, St. Louis, MO, USA), ethylene glycol (Fisher Cat # E178-4, Hampton, NH, USA), and sucrose (Fisher Cat # S5-3, Hampton, NH, USA)) at −20 °C until staining or IHC was performed.

Fluoro-Jade C (FJ-C; Histo-Chem, Jackson, AR, USA; cat# 1FJC), a marker for degenerating neurons and axons [27], was used to stain tissue sections according to the manufacturer’s directions. Briefly, sections were flat-mounted on positively charged slides, washed, incubated in 0.06% potassium permanganate for 5 min, washed, incubated in 0.0001% FJ-C solution (diluted in 1% acetic acid) for 5 min. After staining, slides were air-dried in the dark. Slides were stored without coverslips in a slide box and imaged immediately to reduce background severity.

### 2.4. Immunofluorescence

Primary antibodies used for immunofluorescence were polyclonal rabbit anti-glial fibrillary acidic protein antibody (GFAP 1:2000; DAKO, Santa Clara, CA, USA; cat# Z0334; RRID AB_10013382) and ionized calcium-binding adaptor molecule 1 (Iba-1 1:2000; Synaptic Systems, Goettingen, Germany; cat# 234003, RRID AB_10641962), both at 1:2000 dilution (Table 1). The tissue was incubated in Cy3 secondary antibody (1:500; see Table 1) for 1 h at room temperature before being mounted onto slides and coverslipped with Gelvatol, as we have previously described [14]. As discussed previously, increased GFAP immunoreactivity was measured as an indicator of astrogliosis; IBA-1 labels microglia; soma area and perimeter were used as measures of changing morphology associated with microglial activation [13]. For detailed procedures, see Hetzer et al., 2021 [14]. Table 1 shows more detailed antibody information.

### 2.5. Image Analysis

GFAP and IBA-1 slides were photographed using an Axio Imager Z1 microscope with an Apotome (Leica Microsystems, Buffalo Grove, IL, USA). All slides were photographed using the same exposure and magnification between treatment conditions within each time point. FJ-C slides were imaged on a Nikon C2 Plus Confocal Microscope (Nikon Corporation, Melville, NY, USA) at identical exposure and fluorescence intensity/color settings so that accurate mean fluorescence could be compared between groups. A blinded observer took all pictures. Image analysis and quantification of mean fluorescence intensity were also performed by a blinded investigator using ImageJ software [28] for GFAP and FJ-C. IBA-1 cell soma area and perimeter were measured using Nikon Elements Analysis software (Nikon, Melville, NY, USA).

### 2.6. Statistical Analysis

We performed statistical analysis using the SigmaPlot 14 software package (Systat, San Jose, CA, USA). Alpha was set at 0.05. Within individual analyses, where n did not equal that reported in the cohort numbers above, this was due to loss of tissue, inability to measure fluorescence due to background, removal of a significant outlier (Grubbs test for outliers *p* < 0.05; https://www.graphpad.com/quickcalcs/Grubbs1.cfm (accessed on 10 December 2020)), or the addition of the second 30 DPI cohort. Occasionally, if a significant outlier was found, and its removal from the analysis resulted in no change in statistical outcome, it was retained on the graph for the sake of transparency (e.g., Figure 5A 14 DPI sham). Weight data were first normalized as percent of pre-injury weight, then analyzed using 2-way repeated-measures ANOVA (with injury and time after injury as independent variables). Data are reported as mean and SEM, except where specified otherwise. Post-hoc testing was performed using the Holm–Sidak post-hoc method. Because we did not plan to compare time points and because histologic staining was performed for each time point separately (leading to significant changes in background staining between tissue from each time point), we did not compare directly between time points. We analyzed planned comparisons with Student’s *t*-tests within each time point and reported as mean and SEM in figures. If normality testing failed, Mann–Whitney Rank Sum Tests were used, and those data are reported as the median and interquartile range (see Table 2, Table 3 and Table 4 for test statistics).

After analysis of FJ-C, we noted that FJ staining in TBI groups did not always appear normally distributed for several regions of interest (ROIs) examined. Therefore, we performed post-hoc analyses for normality (i.e., Shapiro–Wilk normality tests and Kolmogorov–Smirnov tests) and graphed the frequency distributions whenever normality failed. After confirming a bi-modal distribution, we split TBI groups into high-staining and low-staining mice using a median split. We then used Student’s *t*-tests between these groups and compared low and high to sham. While we also noted distributions in a few ROIs for GFAP and IBA-1 that appeared similar to these bimodal groups by visual inspection, these analyses did not fail tests for normality, and so were not included.

## 3. Results

### 3.1. Weight, Righting, and Seizures

Mortality was low with only 4 out of 88 mice dying after injury (cause of death appeared to be prolonged apnea, as we have previously reported [13]). There were no skull fractures or other overt physical injuries on or inside the skull. Pre-injury, mice weighed between 18.1 and 24.2 g (M = 20.97, SD = 1.31) and weights between groups were not significantly different on TBI day 0 (*p* = 0.38). There were main effects of injury (F_1,636_ = 24.9, *p* < 0.001) and DPI (F_8,636_ = 32.3, *p* < 0.001) as well as an interaction (F_8,636_ = 2.2, *p* = 0.03). Post hoc analyses revealed significantly lower weights in the TBI group at all time points after injury except 11 and 16 DPI (Figure S1A). Righting times for TBI mice ranged from 1.17 to 21.16 min (M = 5.26, SD = 4.02). Starting weight did not correlate with righting time after injury (r = 0.04, *p* = 0.81; Figure S1B). Some mice showed overt signs of convulsions/generalized tonic-clonic seizures after injury, but these did not correlate with starting weight (r = −0.2, *p* = 0.22; Figure S1C) or righting time (r = 0.18, *p* = 0.25; Figure S1D).

### 3.2. Histology

We examined several regions of interest throughout the cortex, diencephalon, tectum, and brainstem for signs of degeneration and gliosis utilizing FJ-C (statistics in Table 2), GFAP (statistics in Table 3), and IBA-1 (statistics in Table 4) staining. For this study, regions of interest were limited to the visual/optic system. Regions included the optic tract (OT) and its direct projections the dorsal lateral geniculate nucleus (dLGN), ventral lateral geniculate nucleus (vLGN), suprachiasmatic nucleus (SCN), superior colliculi (SC), nuclei of the accessory optic tract—the dorsal (DTN) and medial terminal nuclei (MTN)—the accessory optomotor nucleus (AON), and Edinger-Westphal nuclei (EW). We also investigated multi-synaptic targets including the brachium of the superior colliculus ([BoSC] also includes some direct OT axons), nucleus of the optic tract (NOT), visual cortex (VC), and regions that integrate motor coordination of vision—the substantia nigra *pars reticulata* and caudoputamen (see Figures S2 and S3 for anatomy). For more information on the anatomy of these regions and justification of our choices see Giolli et al. for the accessory optic system [29]; other regions controlling eye movements [30,31]; comprehensive retinofugal projections [32]; superior colliculi division [33], visuo-motor circuit [34,35]; and NOT [36]. Outside these regions, there was somatic staining only in the hippocampus, which we have reported previously [13].

**Figure 2:**
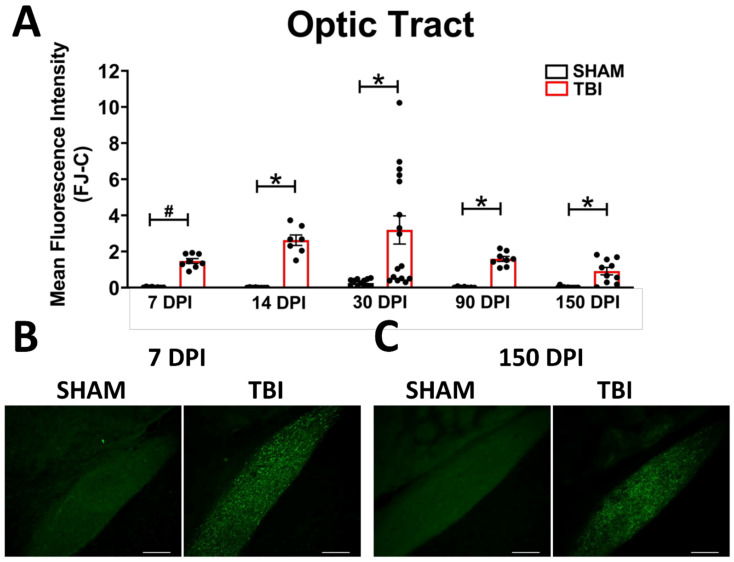
Degeneration in this model begins in the optic nerve and propagates through the optic tract throughout the time course. (**A**) There is significant degeneration (FJ-C staining) in injured mice at all time points compared to sham. Representative images of degeneration early (**B**) and chronically (**C**) after TBI were taken at 20× magnification with a scale bar representing 100 µm. * *p* < 0.001, # *p* < 0.05 for sham vs. TBI within a time point.

**Figure 3:**
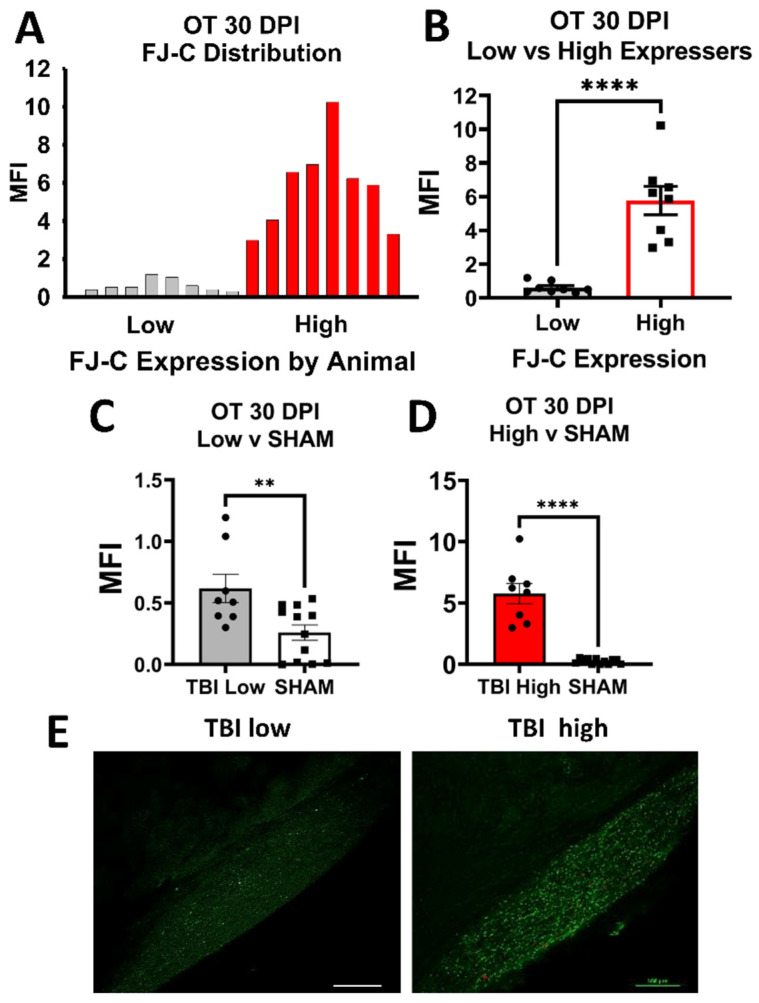
FJ-C expression showed distinct bi-modal characteristics in the OT at 30 DPI. (**A**) We plotted a frequency distribution of FJ-C MFI in TBI mice only and saw two clear distributions of “low” and “high” FJ-C expression. (**B**) These two groups were significantly different from each other potentially indicating inherent differences in recovery from, or susceptibility to, injury in the model of optic axonal injury. (**C**) Low FJ in TBI mice, however, was still significantly high than sham-injured mice (**D**) as were high FJ-C expressing mice. (**E**) We can clearly see little to no degeneration in the low group compared to the high with these representative photomicrographs taken at 20× magnification. Scale bars represent 100 μm. MFI: Mean Fluorescence, OT: Optic Tract, Intensity, ** *p* < 0.01, **** *p* < 0.0001.

**Figure 4:**
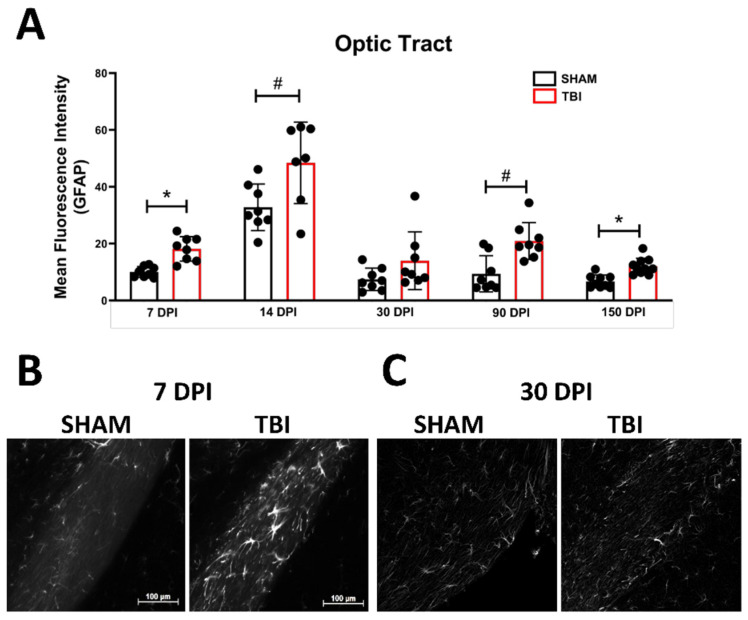
Astrogliosis in the optic tract is marked by early activation, recovery, and subsequent re-activation after TBI. GFAP immunofluorescence (MFI) is significantly increased in the optic tract at all time points except 30 DPI (**A**). (**B**) Representative images of TBI vs. sham mice 7 DPI reveal not only brighter GFAP but also more astrocytes (not quantified) compared to similar levels of both 30 DPI (**C**). Representative photomicrographs were taken at 20× magnification with a scale bar representing 100 µm (applies to all panels). * *p* < 0.001, # *p* < 0.05 for sham vs. TBI within a time point.

**Figure 5:**
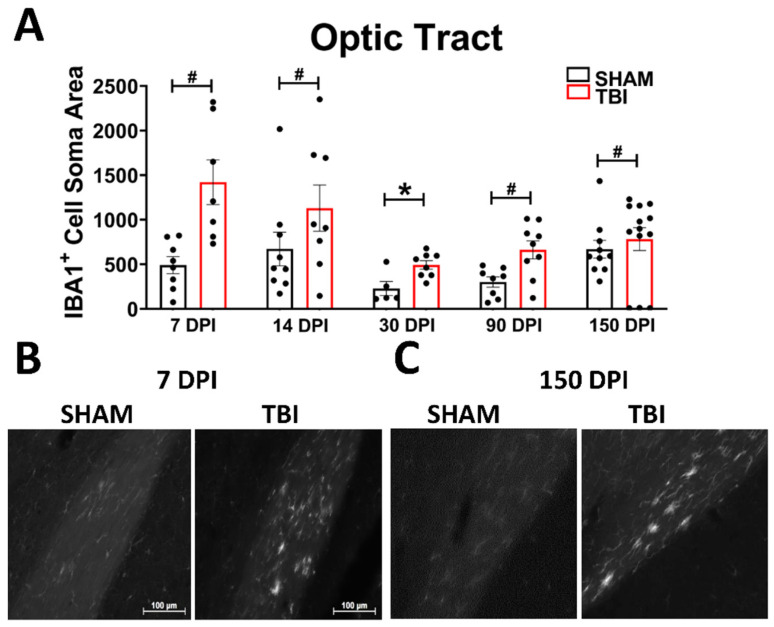
Microglial morphology is persistent in the optic tract. As ameboid microglia represent an immune response and phagocytosis of debris (in this case axonal), we examined the soma size of IBA-1 positive cells in the OT at all time points (**A**). The spherical, bright, ameboid cells can be seen at 7 DPI (**B**) and 150 DPI (**C**). Representative photomicrographs were taken at 20× magnification with a scale bar representing 100 µm (applies to all panels). * *p* < 0.001, # *p* < 0.05 for sham vs. TBI within a time point.

***Optic Tract.*** There was significantly increased FJ-C staining in injured mice at all five-time points (Figure 2A–C; Table 2). At 30 DPI we saw high and low-expression of FJ-C in TBI mice. We confirmed a bi-modal distribution within the TBI group at 30 DPI (Figure 3A) and found significant differences between these groups (*p* < 0.001; Figure 3B). The low expressing FJ mice still presented with significantly higher FJ than sham (*p* = 0.008; Figure 3C) as well as high expressers versus sham (*p* < 0.001; Figure 3D,E). Increased GFAP immunofluorescence (i.e., astrogliosis) was present in the optic tract 7, 14, 90, and 150 DPI (Figure 4A–C; Table 3; Figure S3B). Microglial IBA-1 staining followed the same pattern as FJ-C with significantly larger somata in the OT at all time-points after injury in TBI mice (Figure 5A–C; Table 4; Figure S3C). We also measured soma perimeter, which produced nearly identical results for this and subsequent ROIs (data reported in Table S1).

***Lateral Geniculate Nucleus.*** In the dorsal LGN there was increased axonal degeneration 7, 14, 30, and 90 DPI (Figure S4A,D; Table 2). There was no longer a significant difference between sham and TBI mice 150 DPI; however, there is a significant difference between high and low expression in injured mice (*p* = 0.005; Figure S5I,L) and low FJ-expressing mice were no longer different than sham (*p* = 0.2; Figure S5K). Despite overall increases in FJ in the TBI group 30 and 90 DPI, the TBI group significantly split 30 DPI (*p* < 0.001; Figure S5A,B) and 90 DPI (*p* < 0.001; Figure S5E,F), but low FJ-expressing mice were only not different from sham 90 DPI (*p* = 0.8; Figure S5G). In the ventral LGN, there was significant degeneration in injured mice 7, 14, 30, and 90. DPI 150 was not significantly different between groups (Figure S6A,D), and, again high and low expressers were significantly different from each other (*p* = 0.006; Figure S7I,J), low was similar to sham (*p* = 0.1; Figure S7K), and high was significantly higher than sham (*p* = 0.01; Figure S7L). This split also occurred 30 DPI (*p* = 0.004; low vs. sham *p* = 0.8; high vs. sham *p* < 0.001; Figure S7A–D) and 90 DPI (*p* < 0.001; low vs. sham *p* = 0.56; high vs. sham *p* = 0.001); Figure S7E–H). Additionally, degeneration is not different between the dLGN and vLGN at any time point (Table S2).

In the dLGN, there was no significant difference in GFAP expression in astrocytes between injured and sham mice 7, 14, or 90 DPI (Figure S4B,E; Table 3). There was increased astrogliosis in TBI mice in the dLGN 30 and 150 DPI. The pattern of astrogliosis is the same in the vLGN with no significant effects of TBI in mice 7, 14, or 90 DPI, while there are delayed increases of 30 DPI and chronic resurgence 150 DPI (Figure S6B,E). There are no statistically significant changes to microglia at any time in the dLGN (Table 4); however, a few amoeboid microglia can be found at various time points (see Figure S4C,F). The vLGN shows a similar pattern of microgliosis as it does for astrogliosis (Table 3 and Table 4 respectively) with delayed worsening in the TBI group until 30 and 90 DPI, with subsequent declines in microglial activation. Seven DPI and 14 DPI there were no significant differences between sham and TBI, but, again, there are a few amoeboid microglia present by 14 DPI (Figure S6C,F).

***Suprachiasmatic Nucleus.*** We previously showed no changes to the SCN in our TBI model 7 or 30 DPI [12,37] but at later time points we see differences arise in this direct hypothalamic target of the optic nerve. There was no degeneration visible at any time point—somatic nor axonal (Figure S8A, Table 2). GFAP expression was significantly higher in TBI animals at our latest time point 150 DPI (Table 3). GFAP staining intensity was not different at any other time point (Figure S8B,D). Perhaps even more intriguing, microglial soma sizes significantly increased in TBI mice in the SCN 14 DPI right between previous time points examined and long before astroglial responses (Table 4). No significant microglial activation was observed in TBI mice 7, 30, 90, or 150 DPI (Figure S8C,E). IBA-1 perimeter analyses resulted in the same significance or insignificance as their respective area analyses (Table S1).

***Superior Colliculi.*** Degeneration is limited to the superficial input layers of the superior colliculi with no positive FJ-C staining in the deep output layers. Degeneration was significantly higher in TBI mice at all time points (Table 2). See Figure S9A,D for data and selected images, and Figure S3G for subdivisions of the SC. Interestingly, only 30 DPI FJ staining intensity was bimodal (low vs. high *p* < 0.001; low vs. sham *p* = 0.06; high vs. sham *p* = 0.001; Figure S10). As in the OT, there is significantly increased astrogliosis in TBI mice 7 DPI (Table 3). However, while the OT showed possible improvement in astrogliosis 30 DPI, this improvement arises 14 DPI in the superior colliculi and returns for all following time points (Figure S9B,E). Inflammatory microglial states were only significantly present in TBI superior colliculi 150 DPI (Figure S9F, Table 4), but in looking at the images taken at each time point there were at least a few amoeboid IBA-1 positive cells at all time points and there is a clear difference in microglial morphology more indicative of the activating states (Figure S9C,F). Soma area measured 7, 30, 90, and 150 DPI showed no increased microglial soma size, and these results were consistent with perimeter changes (Table S1).

***Accessory Optic Tract Nuclei*.** We found punctate FJ-C staining in both dorsal and medial terminal nuclei. Degeneration was significantly increased in the DTN at all time points (Figure S11A,D; Table 2) and high vs. low expression was bi-modal and significantly different at 30 DPI (*p* = 0.002; low vs. sham *p* = 0.04, high vs. sham = *p* < 0.001; Figure S12). The MTN also showed increased FJ-C at all time points except 150 DPI (Figure S13A,D). A bi-modal distribution arose at 7 (low vs. high *p* = 0.008, low vs. sham *p* = 0.06, high vs. sham = *p* < 0.001) and 30 DPI (low vs. high *p* = 0.03, low vs. sham *p* = 0.004, high vs. sham = *p* = 0.004; Figure S14). Similar to the superior colliculi rather than the optic tract, the dorsal terminal nucleus of TBI mice is significantly more astrogliotic than sham at all times except 14 DPI (Figure S11B,E; Table 3). The MTN, on the other hand, shows the possible recovery of both 14 and 30 DPI with early increases in activation 7 DPI and later return 90 and 150 DPI (Figure S13B,E). Inflammatory microglial morphology in the DTN shows persistently amoeboid structure at all time points (Figure S11C,F; Table 4). The MTN, however, never shows significant differences between groups (Figure S13C,F).

***Brainstem Optic Nuclei.*** Nuclei involved in pupillary control and eye muscle coordination also receive input from RGCs; thus, it was also important to examine these regions despite previous literature finding no differences in the accessory optomotor nucleus [8]. Specifically, we examined the Edinger–Westphal Nuclei and Accessory Optomotor Nuclei, but there was no degeneration at any time-point in either region (Figure S15D). We also saw no astroglial response in these regions with little to no positive GFAP staining present in either sham or TBI tissue; thus, we could not analyze GFAP intensity in these regions. There were also very few microglia present in both the Edinger-Westphal and accessory optic nuclei, which resulted in a low n for some of our analyses. Nonetheless, soma area and perimeter analyses revealed no significant differences at any time in either region (Figure S15A–C).

***Brachium of the Superior Colliculi.*** FJ-C staining was evident in this region 7, 14, 90, and 150 DPI. Thirty DPI there was no longer a significant difference between sham and TBI mice, and most mice showed no positive staining while others presented with a few bright punctate areas (Figure S16A,B; Table 2). Unexpectedly, astrogliosis in this region followed an almost opposite pattern with significant differences in MFI in TBI mice at only 30 and 150 DPI and no significant differences 7, 14, or 90 DPI (Figure S17A,B; Table 3). IBA-1 soma increases also arose in a different pattern with significant differences at 14 and 150 DPI (Figure S18A,B; Table 4). It is possible that microglial activation differences 30 DPI were obscured as this white matter tract is thin in coronal sections and circular amoeboid characteristics appear more elongated supporting our significant finding in soma perimeter but not area 30 DPI (Table S1). There were no differences in IBA-1 soma area 7, 30, or 90 DPI.

***Nucleus of the Optic Tract.*** Unlike most regions examined, positive FJ-C staining does not arise in the nucleus of the optic tract until two weeks after injury, after which it remains significantly worse than sham at all time-points (Figure S16A,C; Table 2). In the NOT, there was a significant increase in GFAP fluorescence intensity in astrocytes in TBI mice 7 or 30 DPI (Table 3). There were no significant increases 14, 90, or 150 DPI (Figure S17A,C). Microglial activation in TBI mice 7, 14, 30, 90, and 150 DPI was also not significant (Figure S18A,C; Table 4).

***Visual Cortex.*** There was no positive FJ-C staining in the visual cortex (Figure S19A), but there is a delayed glial response. Increased GFAP intensity in TBI mice was present at only 30 DPI and 150 DPI but not 7 DPI, 14 DPI, or 90 DPI (Figure S19B,D; Table 3). This is particularly interesting for three reasons. First, 30 DPI is a time point when astrogliosis in the OT is alleviated. Second, there is another split in TBI mice 30 DPI with very little spread between high vs. low groups, while at 150 DPI all animals have increased astrogliosis compared to shams. Third, positive GFAP staining 30 DPI is not only more intense but also shows very different morphological profiles of the astroglia that we do not see at any other time point including 150 DPI. Finally, while there were no significant differences in IBA-1 soma area between sham and TBI mice in this time course (Figure S19C,E; Table 4), the soma perimeter was significantly increased in TBI mice 30 DPI (Table S1). As was seen in the SC, there is a discernable difference between sham and TBI microglial morphology in terms of their processes (Figure S19C).

***The Visual Corticostriatal Loop.*** Higher-order processing and motor coordination of vision also involves the caudoputamen and substantia nigra *pars reticulata* which receive projections from and project to the SC. While there was no positive FJ-C staining at any time point in the caudoputamen (Figure S20A), the tail of the caudoputamen displayed delayed increases in astrogliosis 90 and 150 DPI with no significant differences at acute or subacute time points 7, 14, or 30 DPI (Figure S20B,D). There is also both early (7 DPI) and delayed (90 DPI) microglial response with no differences subacutely (14 or 30 DPI) or chronically (150 DPI; Figure S20C,E). There was also no degeneration in the SNR (Figure S21A), but there was increased astrogliosis 90 and 150 DPI (Figure S21B,D) possibly indicating that glial cells are communicating in a slower manner, but in the absence of neuronal cell death requiring long periods of time following TBI. There is, however, no microglial response in this region (Figure S21C,E). See Table 3 for GFAP and Table 4 for IBA-1 statistical reports.

## 4. Discussion

In the current study, we show that the adolescent murine visual system is chronically and dynamically vulnerable to a single, mild blunt head injury. Following blunt head trauma, adolescent male mice present with chronic axonal degeneration (visible through punctate FJ-C staining) in the optic nerve to lateral geniculate nuclei, superior colliculi, and into the accessory optic system. We show the potential presence of multi-synaptic signaling from axonal degeneration through the brachium of the superior colliculus to the nucleus of the optic tract despite the absence of neuronal death. Additionally, we show evidence for a critical period between 14–30 days after the injury when the visual system may be recovering/remodeling. This is supported by variable FJ-C accumulation within the TBI group starting 30 DPI and, in some regions, continuing through the duration of the time course.

We also show chronic differences in gliosis patterns 14–150 DPI that, to our knowledge, have not yet been reported. While not as consistent across the five-time points examined as degeneration, gliotic response to injury appears to be more pervasive in that changes arise at some points along our time course in every region examined (save the brainstem). This may indicate that glia can respond to optic nerve injury even in regions with no detectable degeneration. Moreover, this multi-synaptic glial communication could mean that degeneration may arise in cortical/secondary regions given a longer period of time after TBI. Our IBA-1 data also suggest that neuroinflammation, in at least some cases, is a slow, potentially progressive process that can arise as late as 150 days after injury (e.g., the superior colliculi and visual cortex).

Many of our findings are in line with the slow Wallerian degeneration (WD) that occurs after axonal injury in the central nervous system (CNS). WD progresses through mechanisms separate from apoptotic cell death, ref. [38] can occur without causing neuronal loss, ref. [39] and usually does not allow regeneration of lost axons/neurons in the CNS. If repair is possible under WD mechanisms/processes, it typically requires months to years following axotomy or TAI [17,40]. In a longitudinal study for 13 weeks (84 days) after optic nerve stretch, authors showed persistent dieback of intact nerve fibers in the optic tract, as well as continually decreasing myelination and evident Wallerian pathologies [41]. Our data are consistent with this possibility, and we suggest that, later after injury, chronic Wallerian degeneration may begin to slow (such as in regions like the medial terminal nuclei and LGN where Fluoro-Jade staining is no longer significantly present 150 DPI). It is also possible that degenerating nerve fibers may be able to initiate additional degeneration long after initial injury and axon dieback. Our previous data support continuing retinal ganglion cell loss as a result of optic nerve injury up to 30 DPI, but we have yet to analyze retinal samples past this time point. Depending on the injury type and severity, studies utilizing diffuse TAI fluid percussion [42], ultrasound [43], blast [44,45] or optic nerve crush/stretch models [46,47,48] disagree on the time course or presence of RGC death ranging from no RGC loss or showing persistent dieback up to 3 months post injury. Furthermore, recent chronic human studies show that the retina progressively thins up to 35 years after injury implying long-term RGC death [15], which will be crucial to characterize in future studies of TON.

TAI studies exploring the causes of slow WD suggest that residual myelin debris and incomplete clearance of digestion chambers containing dead axon segments could be the driving factor for chronic WD and persistent/ongoing degeneration. Our FJ staining with its punctate characteristics throughout the optic tract and projection targets, which persists through 150 DPI may be labeling this residual myelin debris, supporting the notion of prolonged lack of debris clearance. Additionally, support for incomplete debris removal could come from our glial data. Both astrocytes and microglia can perform phagocytic clearance of cellular/axonal debris [49,50,51], and this ability, at least in microglia, can become dysregulated after experimental TBI [52]. The lack of amoeboid/phagocytic microglia in regions high in FJ-C staining, like the dLGN and MTN, and their delayed morphological change in most other regions outside the OT could indicate that glia fail to clear axonal debris up to 150 DPI in mice. Direct measures of microglial activity and function, however, are needed to confirm this idea.

Further, the axonal degeneration seen in grey matter and secondary regions (i.e., regions not directly connected to the OT) along with delayed glial responses indicates potential trans-synaptic WD following TBI in regions like the VC. However, we should note that we did not see degeneration in the single-synaptic region of the suprachiasmatic nuclei, nor the trans-synaptic deeper brainstem projection nuclei (EW/AON) or cortical regions (VC). While this lack of cortical and SCN degeneration is consistent with our previously published results [12,14], it is interesting that brainstem projections are also unaffected by this injury. The Edinger–Westphal nucleus is a compelling region for examination after optic nerve injury due to its proximity to the oculomotor complex (aka, AON, which is also unaffected) and the fact that damage to the retina and optic nerve is often reflected in the loss of proper pupillary function [53]. However, the connection between the optic nerve and EW is multi-synaptic. Moreover, we proposed in our previous work that the lack of SCN response to injury may be because optic nerve projections here are made up primarily of axons from the more resilient intrinsically photosensitive retinal ganglion cells (ipRGCs). ipRGCs respond to light cues in the retina, and project primarily to SCN. They also project to the pretectal nuclei, which signal to the EW for pupillary dilation [54].

It also seems that roughly one month after injury, mice appear to split into at least two groups: one with high FJ-C staining and one with levels near sham mice. These splits are seen 30 DPI in the optic tract, LGN, SC, and AOS; 90 and 150 DPI in the LGN (potentially mirrored in glial response); and as early as 7 DPI in the MTN. Despite continued degeneration in the OT at 30 DPI, the significant reduction in a subset of mice at 30 DPI in almost all direct projection regions may indicate that 30 DPI is the critical time at which a decision is made to protect the brain from further effects of injury or not. This is particularly interesting when considering the fact that we saw low FJ staining in projection regions 90 and 150 DPI while the OT remained high, indicating that the OT at 30 DPI may dictate how the downstream targets respond over time. It is important to note that our 30 DPI time point had a higher number of mice than other times due to the addition of the replacement cohort. It may be that this increased number of subjects allowed more statistical power to detect a divergence between low-degeneration groups and high-degeneration groups. Thus, larger cohorts may be necessary to determine exactly when a subset of mice begins to show resiliency to injury. However, although the other 4 time points had lower n’s, we still saw bi-modal distributions in multiple regions at 7, 90, and/or 150 DPI indicating that the split distributions were likely not the result of two different cohorts. Additionally, the high/low groups at 30 included animals from both the original cohort and the added second cohort. We were unable to follow individual mice across time points to track individual differences due to the invasive and terminal nature of histologic measures. Future research is needed to determine whether this is due to individual differences or the presence of an unknown factor promoting glial clearance of axonal debris.

WD also includes recovery and delayed progression of axonal degeneration, and involves multiple cell types in the tissue surrounding injury [24] including both astrocytes and microglia [55,56]. Tissue responses to injury include the production of “astroglial scars,” which can both promote regeneration [57,58,59] and prevent axon regrowth/repair [57]. Glial scars involve both astrocytic and microglial pro- and anti-inflammatory responses to injury [56,60,61], and glia direct CNS responses to injury and disease [56]. For example, microglia-astrocyte coordination is likely required for axon survival and regeneration where lack of a microglial response shifts astrocytes to a pro-inflammatory state in a mouse model of demyelination [62]. Yet, studies probing the activity of glial cells during regeneration after traumatic axonal injury, in particular, are lacking [20,63]. Our data suggest that astrocytes and microglia respond differently in relation to each other within regions at each time point, such as in the LGN where astrocytes are more consistently reactive compared to microglia, with no microglial response in the dLGN vs. vLGN. This also appears true in secondary projection regions like the NOT where there is no microglial morphology change despite early increased GFAP expression and FJ-C staining, or in the BoSC where the critical/recovery period we see arises later than in the OT or LGN. This non-parallel response between glial cell types may explain why studies focusing on microglia or astrocytes alone have only been partially successful [64,65].

Our data argue not only for the co-activation of glial cells in a region-dependent manner but also in a time-dependent manner. While temporal coordination/dependence of microglial and astrocyte activation is not well understood, one study showed that microglia are dysregulated shortly after brain injury with possible recovery in a time-dependent manner [52]. Although we did not directly examine microglial signaling pathways, our data are consistent with the possibility of reduced inflammatory response 14–30 DPI. We also support the notion of persistent glial dysfunction. For example, ameboid microglia and reactive astrocytes persist throughout the time-course in the optic tract and LGN, but degeneration remains. Moreover, in the SC, where microglial changes are not seen until 150 DPI, this could indicate a delayed spread of inflammatory signals, delayed response of microglia despite inflammatory signals, or even an impaired ability for microglia to sense the inflammation if it is present in these more distal regions. We also report sub-acute and chronic glial activation without degeneration in the visual cortex and corticostriatal loop as well as the concordant appearance of both astrocytes and microglia in predominantly grey-matter regions like the LGN, SC, and VC potentially implicating signaling events we have yet to elucidate.

Not only did one glial type lack activation in some regions, but astrocytes and microglia also sometimes reversed which was significantly active (e.g., microglia are activated earlier than astrocytes in the caudoputamen and SCN, while astrocytes are reactive before microglia change in the SC). These differences may suggest that discovering how and when glial cells communicate after an injury is critical to long-term treatment strategies after axonal injury. At longer time points, we may see microglia beginning to clear the axonal debris. Alternatively, because our 14 and 30 DPI critical periods of reduced glial activation and degeneration are a bit earlier than the 60 DPI time-point examined by Izzy et al. [52], the time-course of the inflammatory response may be slightly different after TAI compared to cortical injury. Future studies would need to follow these processes comparing the different types of injury.

Another aspect of TBI altogether may help explain astrocyte reactivity—cerebrovascular alterations. The CNS response to vascular injury/change after TBI has been well studied [66,67,68,69,70], and astrocytes are known to intimately sense changes to the vascular environment in the CNS (reviewed in [71]). TBI literature agrees that cerebrovascular function is largely impaired after TBI due to an accumulation of reactive oxygen/nitrogen species [66,72], evidence shows that these microvascular changes can persist chronically [68,69], and astrocyte dysfunction after TBI have been correlated with cerebrovascular impairment leading to neuronal dysfunction (reviewed in [73]). Although we saw no hemorrhages in our mice, we did not perform any assays to assess vascular abnormalities, blood-brain-barrier disturbance, or astrocyte-vascular interactions. This would be particularly interesting as it could provide more insight into astrocyte function and response to this injury and may answer some questions about the multi-synaptic response or chronic glial dysfunction.

Although this study was limited in its ability to compare changes between time points due to the nature of our post-mortem endpoints, valuable information was obtained within each time point, and these findings support a need for further investigation into the chronic effects of TON/TAI. Additionally, we cannot fully rule out the potential cohort effects that may have resulted in the bi-modal distributions found in FJ-C expression because we could not perform this assay in the same mouse over time. We were also limited in our ability to measure functional outcomes after injury either in visual function or glial responsivity, so future studies should inspect more factors like phagocytic activity of glial cells, vesicular-astrocyte interactions, and retinal cell response to optic nerve injury. Moreover, our lack of degeneration but 14 DPI astrocyte reactivity in the SCN support a need for further characterization of which retinal cells are affected by this injury including ipRGCs, rods, and cones. Despite these limitations, our results are consistent with the chronic nature of TON in human populations and should implore us to better understand these long-term changes to better treat people with TON.

## 5. Conclusions

Our data provide evidence for a window of time in which the optic/visual system may be recovering from a blunt head injury, because TBI and sham mice do not differ at subacute time points (14–30 DPI), outside the OT. Furthermore, there appears to be a subset of mice that have significantly less degeneration after head trauma from 30–150 DPI, and given that there is not a stratification of severity prior to these times (by histologic means), this suggests there may be individuals with improved long-term recovery. We show that chronic visual system changes after head injury are characterized by tissue responses in the brain, in addition to previously reported retinal thinning and RGC loss. These tissue responses include progressive degeneration and/or gliosis in multiple direct and multi-synaptic cortical and thalamic (but not brainstem) optic nerve projection targets, which worsen chronically after an initial period of apparent recovery. Degeneration, however, is not necessarily predictive of gliotic responses. Rather, its chronic presence may simply be the trigger for occasionally delayed inflammatory. This was shown through dynamic glial response to injury that was dependent upon projection region and time after said injury. Overall, these data are significant in that they support a potential timeline for interventions after TBI/TAI in the visual system beyond the acute post-injury phase.

## Figures and Tables

**Table 1:** Antibody Information.

Antibody	RRID	Host Species	Immunogen	Manufacturer	Concentration
GFAP	AB_10013382	Rabbit	Whole bovine GFAP, isolated from spinal cord.	DAKO (Agilent) CAT: Z033401-2 Lot: 00059585	1:2000
Iba-1	AB_10641962	Rabbit	Peptide from the c-terminal sequence from rat Iba-1: PTGPPAK KAISELP	Synaptic Systems CAT: 234003 Lot: 234003/9	1:2000
Cy3 AffiniPure Donkey anti-Rabbit IgG (H + L) conjugated secondary	AB_2307443	Donkey	Gamma immunoglobulins, heavy and light chains	Jackson Immuno Research CAT: 711-165-152 Lot: 79424	1:500

**Table 2:** Statistics for IHC—Fluoro Jade-C.

**Time Point**	**OT**	***p***	**dLGN**	***p***	**vLGN**	***p***	**SC**	***p***
7 DPI	t(14) = −16.94	*p* < 0.001	t(14) = −4.04	*p* < 0.001	t(14) = −5.17	*p* < 0.001	t(11) = −12.46	*p* < 0.001
14 DPI	t(13) = −11.08	*p* < 0.001	U(13) = 0	*p* < 0.001	U(13) = 0	*p* < 0.001	U(13) = 0	*p* < 0.001
30 DPI	U(26) = 18	*p* < 0.001	t(14) =−4.78	*p* < 0.001	U(25) = −39.5	*p* = 0.02	t(26) = −4.12	*p* < 0.001
90 DPI	t(13) = −16.57	*p* < 0.001	t(14) = −0.77	*p* = 0.05	t(13) = −2.47	*p* = 0.03	t(12) = −6.21	*p* < 0.001
150 DPI	t(19) = 6.74	*p* < 0.001	U(15) = 26	*p* < 0.001	t(15) = 0.79	*p* = 0.44	t(15) = −5.60	*p* = 0.002
	**DTN**	***p***	**MTN**	***p***	**BoSC**	***p***	**NOT**	***p***
7 DPI	U(15) = 0.0	*p* = 0.004	t(14) = −3.44	*p* = 0.004	t(14) = −4.69	*p* < 0.001	U(13) = 22	*p* = 0.54
14 DPI	U(14) = 0	*p* < 0.001	U(12) = 0	*p* = 0.001	t(13) = −4.00	*p* = 0.001	t(13) = −3.27	*p* = 0.006
30 DPI	U(15) = 4	*p* = 0.002	U(15) = 2	*p* < 0.001	U(13) = 14	*p* = 0.12	U(24) = 43	*p* = 0.04
90 DPI	t(13) = −4.95	*p* < 0.001	U(14) = 0	*p* < 0.001	t(12) = −5.09	*p* < 0.001	t(13) = −3.28	*p* = 0.006
150 DPI	t(14) = 0.02	*p* = 0.02	U(17) = 38	*p* = 0.89	t(17) = −5.76	*p* < 0.001	U(19) = 19	*p* = 0.02

Test statistics for histological FJ-B data. t indicates a Student’s *t*-test; U indicates that data failed normality and a Mann–Whitney Rank-Sum analysis was performed; *p* represents *p*-values of analyses. Abbreviations: OT: Optic Tract; dLGN: dorsal Lateral Geniculate Nucleus; vLGN: ventral LGN; SC: Superior Colliculi; DTN: Dorsal Terminal Nucleus; MTN: Medial Terminal Nucleus; BoSC: Brachium of the Superior Colliculi; NOT: Nucleus of the Optic Tract.

**Table 3:** Statistics for IHC—GFAP.

**Time Point**	**OT**	***p***	**dLGN**	***p***	**vLGN**	***p***	**SCN**	***p***
7 DPI	t(14) = 5.27	*p* < 0.001	t(14) = −1.56	*p* = 0.14	t(14) = 0.12	*p* = 0.90	t(13) = 1.02	*p* = 0.33
14 DPI	t(13) = −2.65	*p* = 0.02	t(13) = 1.13	*p* = 0.28	t(13) = 1.25	*p* = 0.23	t(13) = 1.11	*p* = 0.29
30 DPI	t(14) = 2.07	*p* = 0.07	t(14) = −4.00	*p* = 0.001	t(14) = −3.64	*p* = 0.003	t(13) = −2.28	*p* = 0.25
90 DPI	t(14) = −3.91	*p* = 0.002	t(14) = −1.76	*p* = 0.10	t(14) = −1.09	*p* = 0.43	t(14) = 1.78	*p* = 0.10
150 DPI	t(17) = −4.49	*p* < 0.001	t(18) = 2.51	*p* = 0.02	t(18) = 3.71	*p* = 0.002	t(18) = 2.51	*p* = 0.02
	**SC**	***p***	**DTN**	***p***	**MTN**	***p***	**BoSC**	***p***
7 DPI	t(13) = −4.12	*p* = 0.001	t(14) = −3.68	*p* = 0.002	t(14) = −3.03	*p* = 0.008	t(14) = −1.43	*p* = 0.17
14 DPI	t(13) = −1.41	*p* = 0.18	t(13) = 0.13	*p* = 0.90	t(13) = 0.95	*p* = 0.36	t(13) = 0.54	*p* = 0.02
30 DPI	t(13) = −3.81	*p* = 0.002	U(14) = 11	*p* = 0.03	U(13) = 21	*p* = 0.46	t(14) = 2.56	*p* = 0.59
90 DPI	t(14) = 2.83	*p* = 0.1	t(14) = −6.63	*p* < 0.001	t(14) = −2.20	*p* = 0.04	t(13) = −2.89	*p* = 0.01
150 DPI	t(18) = −4.51	*p* = 0.002	t(18) = 4.52	*p* < 0.001	t(18) = 5.12	*p* < 0.001	t(18) = 2.40	*p* = 0.03
	**NOT**	***p***	**VC**	***p***	**SNpr**	***p***	**CPu**	***p***
7 DPI	t(14) = −2.94	*p* = 0.01	t(14) = 0.76	*p* = 0.47	t(14) = −0.69	*p* = 0.50	t(14) = −1.24	*p* = 0.23
14 DPI	t(13) = −0.46	*p* = 0.66	t(13) = −0.74	*p* = 0.47	t(13) = 0.43	*p* = 0.67	t(13) = 0.34	*p* = 0.74
30 DPI	t(14) = 2.56	*p* = 0.02	t(13) = −2.28	*p* = 0.04	t(14) = 1.25	*p* = 0.23	t(14) = 0.98	*p* = 0.34
90 DPI	t(14) = −1.94	*p* = 0.07	t(12) = −0.82	*p* = 0.42	t(13) = −3.12	*p* = 0.008	t(13) = −2.85	*p* = 0.01
150 DPI	t(18) = 1.27	*p* = 0.22	t(18) = 2.32	*p* = 0.03	t(18) = 2.16	*p* = 0.04	t(18) = 2.99	*p* = 0.007

Test statistics for histological GFAP data. *t* indicates a Student’s *t*-test; U indicates that data failed normality and a Mann–Whitney Rank-Sum analysis was performed. Abbreviations not included with Table 2: SCN: Suprachiasmatic Nucleus, VC: Visual Cortex; SNpr: Substantia Nirgra pars reticulate; Cpu: Caudoputamen.

**Table 4:** Statistics for IHC—IBA-1 (Soma Area).

**Time Point**	**OT**	***p***	**dLGN**	***p***	**vLGN**	***p***	**SCN**	***p***	**SC**	***p***
7 DPI	t(13) = −3.4	# *p* = 0.005	U(13) = 22	*p* = 0.33	t(14) = −0.86	*p* = 0.486	t(14) = −0.16	*p* = 0.87	t(13) = 0.62	*p* = 0.55
14 DPI	t(12) = −2.69	# *p* = 0.02	t(13) = −0.78	*p* = 0.45	t(12) = −0.89	*p* = 0.39	t(13) = 2.89	# *p* = 0.01	t(13) = 0.01	*p* = 0.90
30 DPI	t(11) = −3.04	# *p* = 0.01	t(14) = −0.58	*p* = 0.58	t(14) = −2.42	# *p* = 0.03	t(13) = −0.08	*p* = 0.94	t(13) = −1.29	*p* = 0.18
90 DPI	t(14) = −4.19	* *p* < 0.001	t(14) = −0.40	*p* = 0.57	t(14) = −2.50,	# *p* = 0.03	t(13) = 0.78	*p* = 0.45	t(14) = −0.8	*p* = 0.44
150 DPI	t(18) = −3.45	# *p* = 0.003	t(18) = −0.44	*p* = 0.70	t(18) = 1.45	*p* = 0.16	t(18) = −0.33	*p* = 0.74	t(15) = −3.78	# *p* = 0.002
	**DTN**	***p***	**MTN**	***p***	**EW**	***p***	**AON**	***p***	**BoSC**	***p***
7 DPI	t(13) = −3.26	# *p* = 0.006	t(13) = −0.68	*p* = 0.51	U(13) = 19	*p* = 0.34	t(13) = −1.62	*p* = 0.13	t(14) = −1.65	*p* = 0.12
14 DPI	t(13) = −4.99	* *p* < 0.001	t(13) = 1.26	*p* = 0.23	t(13) = 0.03	*p* = 0.97	t(13) = 1.23	*p* = 0.24	t(13) = −2.4	# *p* = 0.03
30 DPI	t(13) = 7.79	* *p* < 0.001	t(11) = −1.85	*p* = 0.09	t(10) = −0.21	*p* = 0.84	T(13) = −2.09	*p* = 0.06	t(22) = 1.80	*p* = 0.08
90 DPI	t(13) = −3.26	# *p* = 0.006	t(13) = −0.68	*p* = 0.51	U(13) = 19	*p* = 0.34	t(13) = −1.62	*p* = 0.13	t(8) = −1.76	*p* = 0.06
150 DPI	U(18) = 16	# *p* = 0.01	U(18) = 37	*p* = 0.35	t(16) = 1.32	*p* = 0.20	t(18) = −0.59	*p* = 0.57	t(17) = 3.1	# *p* = 0.006
	**NOT**	***p***	**VC**	***p***	**SNpr**	***p***	**CPu**	***p***		
7 DPI	t(14) = −1.33	*p* = 0.21	t(14) = −0.26	*p* = 0.80	U(14) = 22	*p* = 0.33	t(13) = −0.25	*p* = 0.8		
14 DPI	t(13) = −1.19	*p* = 0.47	t(13) = −0.96	*p* = 0.35	t(13) = −0.32	*p* = 0.75	t(13) = −0.21	*p* = 0.83		
30 DPI	t(18) = −0.49	*p* = 0.63	U = 42	*p* = 0.26	t(12) = −0.26	*p* = 0.79	t(14) = −1.03	*p* = 0.32		
90 DPI	t(7) = 0.81	*p* = 0.36	t(14) = −0.48	*p* = 0.26	U(14) = 22	*p* = 0.33	t(13) = −2.44	# *p* = 0.03		
150 DPI	t(17) = 0.23	*p* = 0.82	t(18) = 0.07	*p* = 0.65	t(18) = −1.38	*p* = 0.18	t(17) = 0.37	*p* = 0.72		

Test statistics for histological IBA-1 soma area analyses. *t* indicates a Student’s *t*-test; U indicates that data failed normality and a Mann–Whitney Rank-Sum analysis was performed. * *p* < 0.001, # *p* < 0.05. See previous tables for abbreviations.

## Data Availability

Data are available from the authors upon request.

## Supplementary Materials

The following are available online at https://www.mdpi.com/article/10.3390/cells10123343/s1, Figure S1: Adolescent mice experience weight loss following TBI; Figure S2a: Regions of Interest; Figure S2b: Regions of Interest continued; Figure S3: Images of Optic Tract (OT); Figure S4: The LGN has a similar pattern of degeneration as the OT that persists throughout the time course; Figure S5: From 30 DPI on, the dLGN shows two distinct populations of mice; Figure S6: The vLGN is affected by TAI in a similar manner as the dLGN save for delayed microglial activation; Figure S7: From 30 DPI degeneration in the vLGN shows two distinct populations of mice; Figure S8: Despite a general lack of cellular disturbance to TAI in the SCN, microglia alone become reactive only at 14 DPI; Figure S9: The input layers of the superior colliculi are not spared from degeneration or glial reactivity after TON, but inflammatory activation is chronically delayed; Figure S10: At 30 DPI degeneration in the SC shows two distinct populations of mice with a low group showing potential resiliency to injury; Figure S11: The white matter tracts of the accessory optic system are also affected by OT TAI chronically; Figure S12: At 30 DPI, mice continue to show the bi-modal distribution of FJ-C reactivity in the DTN of the accessory optic system; Figure S13: The MTN of the accessory optic system shows nearly identical response to TAI as the DTN; Figure S14: The MTN shows the potential for a group of resilient mice acutely and subacutely; Figure S15: Pupillary control regions are not affected by TAI; Figure S16: The BoSC and secondary OT projection site—the NOT—also contain degenerating axons chronically after TAI; Figure S17: Astrocyte reactivity is immediate in the NOT and delayed in the BoSC; Figure S18: Increased microglial somata are only present in the white-matter BoSC and not the NOT; Figure S19: Despite a lack of degeneration, glial cells show increased response to injury in the visual cortex by 30 DPI; Figure S20: The substantia nigra pars reticulate at 90 DPI showed astroglial response to injury; Figure S21: The caudoputamen responds to OT injury staring at 90 DPI until the end of the time course; Table S1: IBA-1 Positive Soma Perimeter Measurements, Table S2. dLGN vs. vLGN FJ-C ANOVAs (No effect of location within LGN).

## Abbreviations

TBI	Traumatic Brain Injury	SD	Standard deviation
TAI	traumatic axonal injury	OT	Optic Tract
iTON	indirect traumatic optic neuropathy	dLGN	dorsal lateral geniculate nucleus
WD	Wallerian Degeneration	vLGN	Ventral lateral geniculate nucleus
O_2_	oxygen	SCN	suprachiasmatic nucleus
DPI	Days post-injury	SC	superior colliculi
IHC	immunohistochemical	DTN	Dorsal terminal nucleus
PBS	phosphate-buffered saline	MTN	medial terminal nucleus
FJ-C	Fluoro-Jade C	AON	accessory optomotor nucleus
GFAP	glial fibrillary acidic protein	EW	Edinger-Westphal nuclei
Iba-1	ionized calcium-binding adaptor molecule 1	BoSC	brachium of the superior colliculus
ANOVA	Analysis of variance	NOT	nucleus of the optic tract
SEM	Standard error of the mean	VC	visual cortex
ROI	Region of interest	CNS	Central nervous system
M	Mean		
