# Supplementary material for: Chronic Histological Outcomes of Indirect Traumatic Optic Neuropathy in Adolescent Mice: Persistent Degeneration and Temporally Regulated Glial Responses
Source: Cells. 2021 Nov 28;10(12):3343. doi: 10.3390/cells10123343 (PMC8699438; doi:10.3390/cells10123343)
Supplement: Supplementary file 1 [file cells-10-03343-s001.zip › cells-1460186-supplementary.pdf]

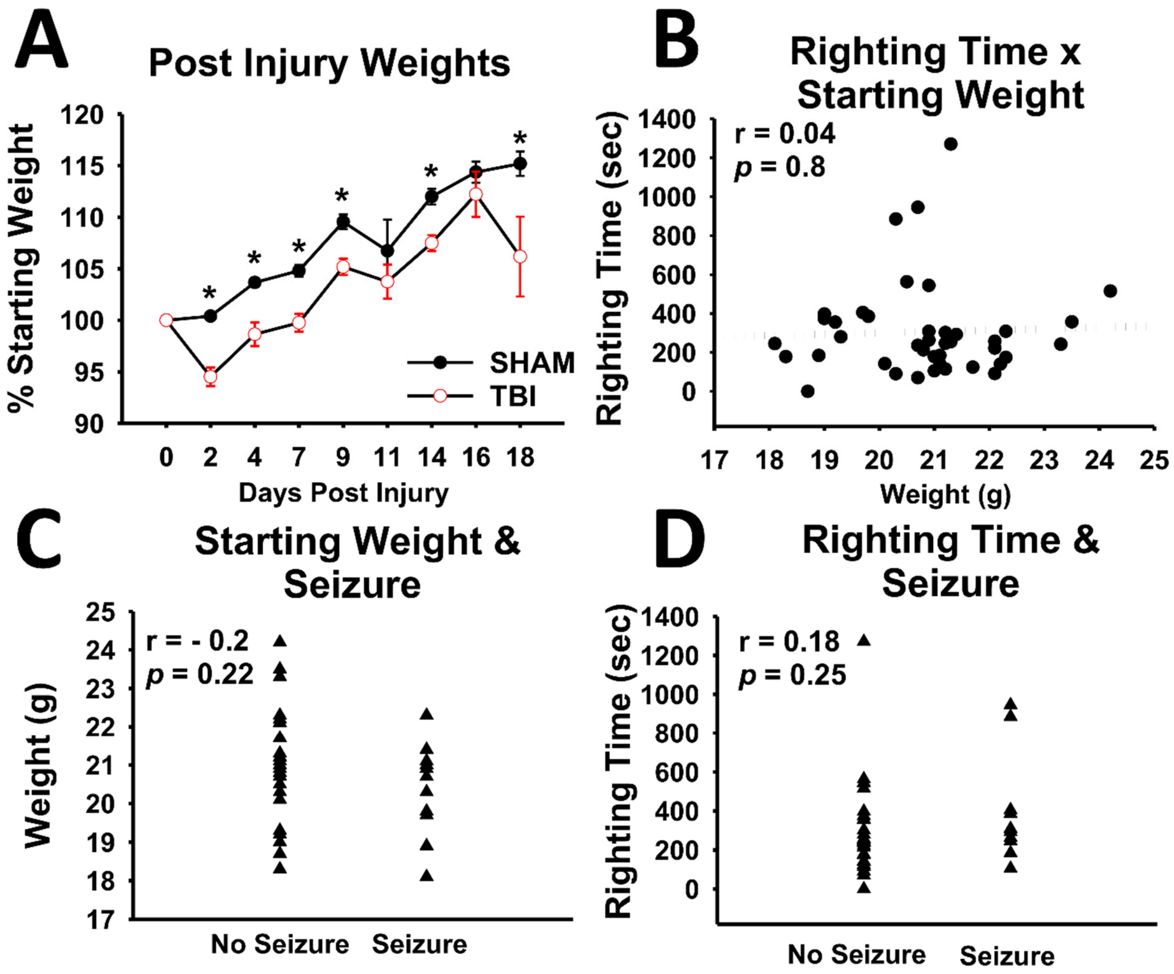

Figure S1. Adolescent mice experience weight loss following TBI. After injury, mice have reduced weight up to 18 DPI at all time points except 11 and 16 DPI compared to non-injured counterparts (A). Righting time, or the righting reflex, was monitored as it is a measure commonly associated with severity of injury. Starting weight is not correlated with righting time (B) or having a seizure after impact (C). Additionally, having a seizure does not impact righting time (D). Mortality was low at 0.8% with 4 of 46 TBI mice expiring shortly after injury (not graphed) in line with this being a mild-moderate injury. g = grams, sec = seconds, * indicates p <0.001; black dotted line = regression line.

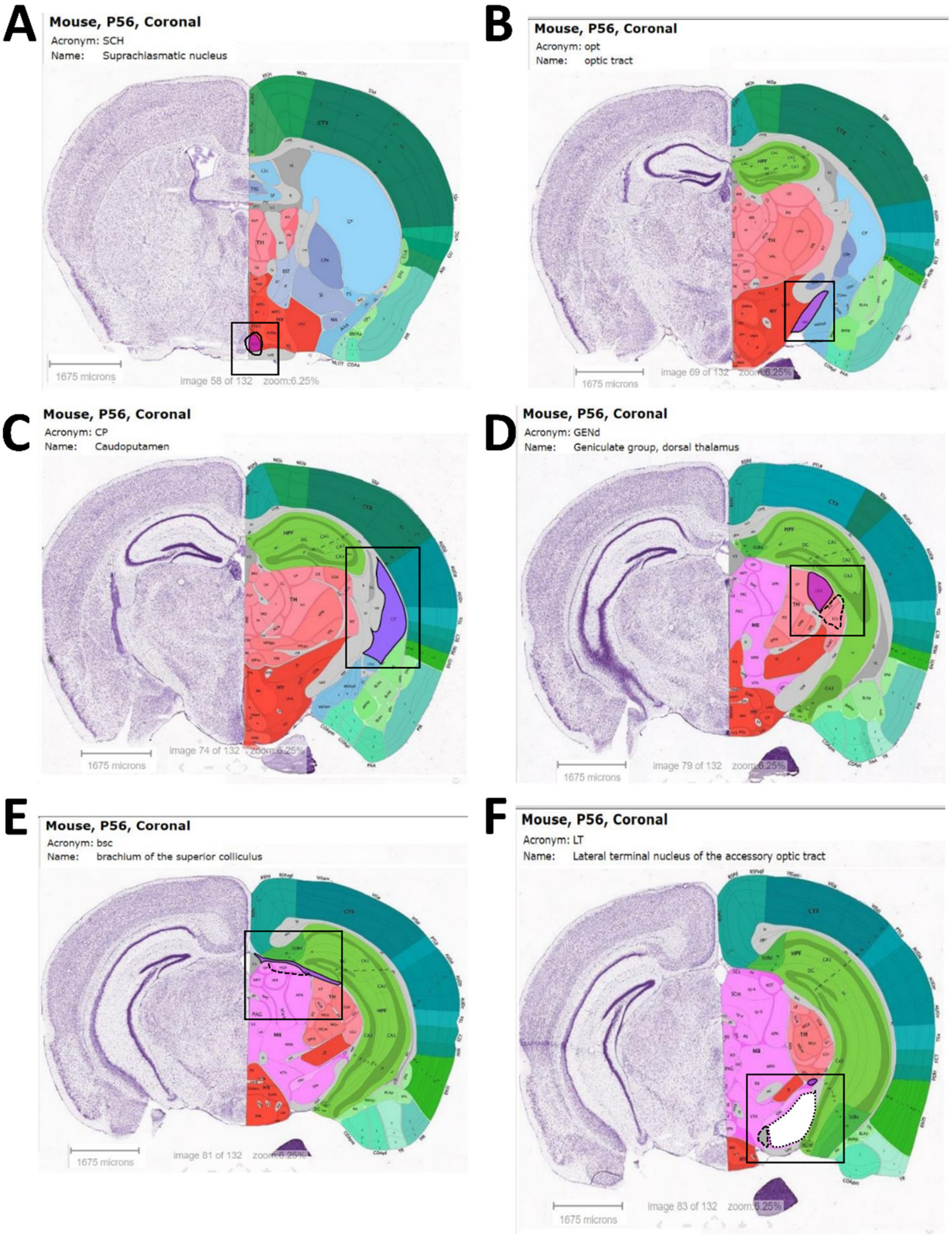

Figure S2a. Regions of Interest. Using the coronal Allan Mouse Brain Atlas as a guide, sections of brain tissue cut at 30 µm at roughly each of the pictured coordinates were imaged and analyzed for (A) Suprachiasmatic Nucleus – black outline, (B) Optic Tract – black outline, (C) CaudoPutamen – black outline, (D) Dorsal Lateral geniculate (black outline) and ventral LGN (dashed black outline), (E) Brachium of the Superior Colliculus (black outline) and Nucleus of the Optic Tract (Dashed outline), (F) Dorsal (aka lateral – see results) Terminal Nucleus (purple with black outline, Substantia Nigra pars reticulata (white with dashed out line), and medial terminal nucleus (grey with dashed outline). Image credit: Allen Institute. Each image shows the image number which can be found in reference to the Mouse, P56, Coronal Atlas (http://atlas.brain-map.org/atlas).

G

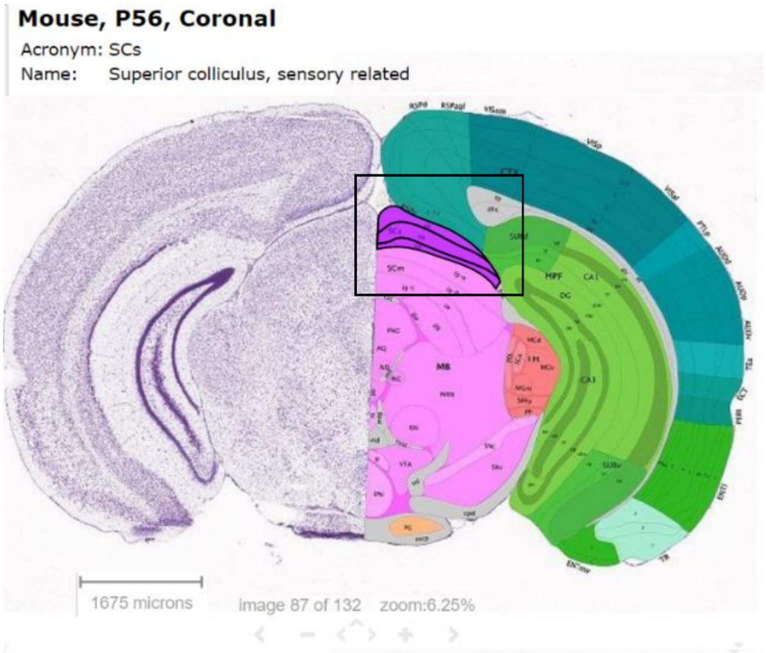

H

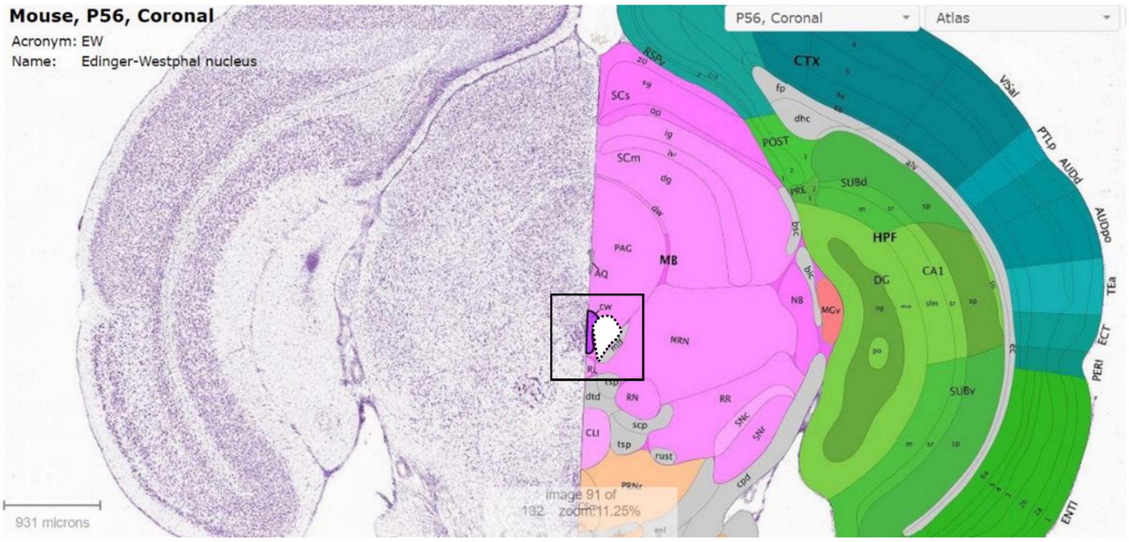

Figure S2b. Regions of Interest continued. Using the Allan Mouse Brain Atlas as a guide sections of brain tissue cut at 30 μm at roughly each of the pictured coordinates were imaged and analyzed for (G) Superior colliculi superficial layers, and (H) Edinger-Wastphal Nuclei (purple with black outline, and optomotor nucleus (white with dashed outline). Image credit: Allen Institute. Each image shows the image number which can be found in reference to the Mouse, P56, Coronal Atlas (http://atlas.brain-map.org/atlas).

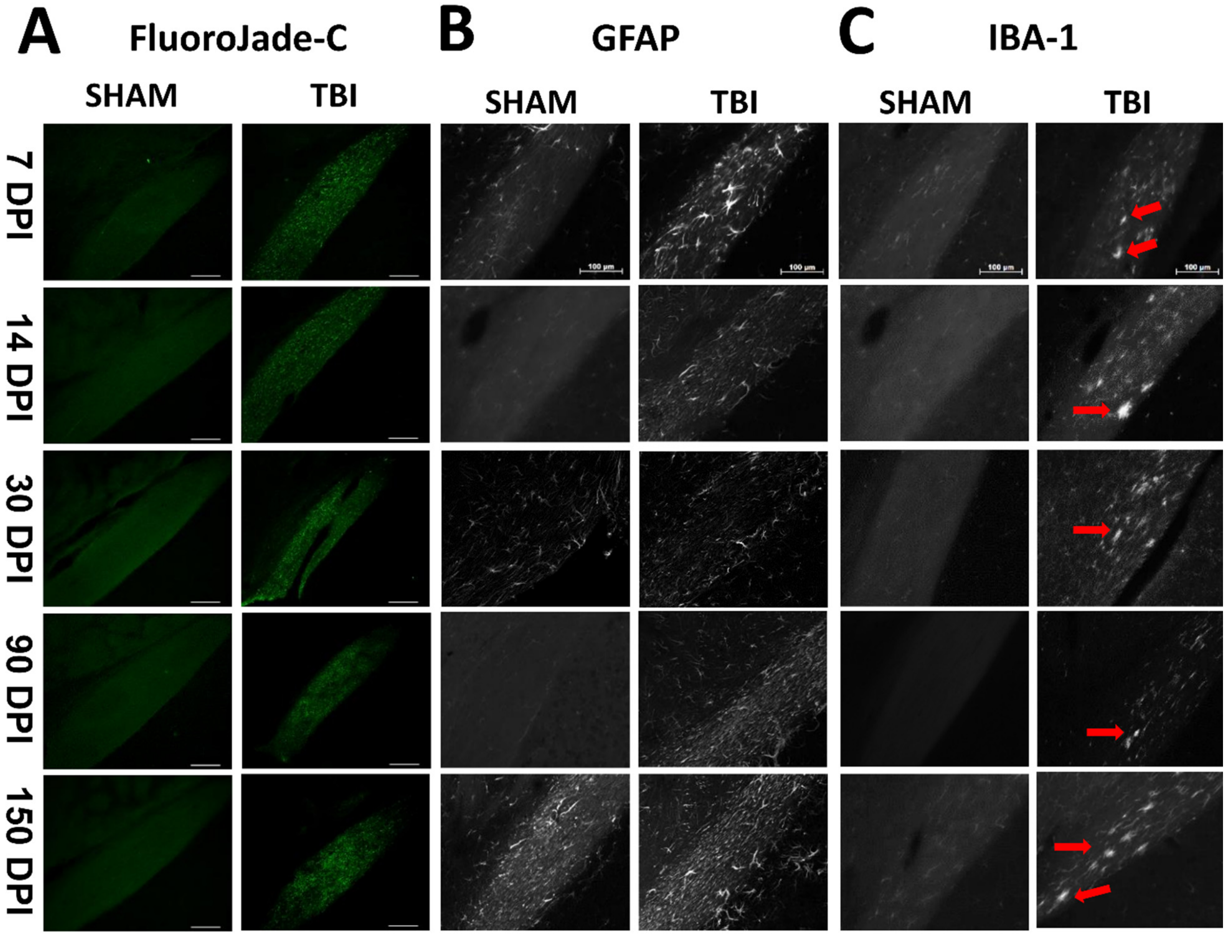

Figure S3. Images of Optic Tract (OT). (A) FluoroJade-C (FJ-C) OT representative images for each timepoint examined. For Sham images background was enhanced in order to visualize structures. For TBI images contrast was enhanced to visualize punctate staining more easily. (B) GFAP OT images for all times points. (C) IBA-1 OT images. Red arrows highlight characteristically larger/ameboid microglial cells. Scale bars indicate 100μm and can each be applied to all images within their respective stains.

Table S1. IBA-1 Positive Soma Perimeter Measurements

Time Point	OT	dLGN	vLGN	SCN	SC	BoSC	NOT	VC
**7 DPI**	t(13)= -2.58 p=0.02	t(14)= -0.20 p=0.84	t(14)=-0.73, p=0.48	t(14)= -0.66 p=0.52	t(13)= 0.49 p=0.63	t(13)= 0.07 p=0.11	t(14)= 1.01 p=0.82	t(14)= 0.16 p=0.87
**14 DPI**	t(12)= -1.69 p=0.12	t(13)= -0.17 p=0.87	U(13)= 59 p=0.78	t(13)= 2.93 p=0.01	t(13)= 0.17 p=0.9	t(13)= 0.29 p=0.77	t(13)= 0.87 p=0.67	t(13)= 1.08 p=0.28
**30 DPI**	t(11)= -2.29 p=0.04	t(14)= -0.62 p=0.55	t(14)=-0.97 p=0.35	t(13)= -0.32 p=0.75	t(13)= -1.37 p=0.19	t(22)= 2.63 p=0.01	t(18)= 0.74 p=0.46	t(21)= 2.84 p=0.006
**90 DPI**	t(14)= -3.45, p=0.004	t(14)= -0.77 p=0.46	t(14)= -2.53 p=0.024	t(13)= -0.66 p=0.52	t(14)= -1.21 p=0.25	t(8)= 0.92 p=0.36	t(7)= 1.47 p=0.15	t(14)= 0.13 p=0.89
**150 DPI**	t(18)= -2.78, p=0.012	U(18)= 118 p=0.34	t(18)=1.69 p=0.11	t(18)= -0.44 p=0.67	t(15)= -3.90 p=0.001	t(17)= 2.99 p=0.004	t(17)= 0.97 p=0.34	t(18)= 0.15 p=0.88
	**DT**	**MT**	**FEF**	**SNpr**	**CPu**	**EW**	**AON**	
**7 DPI**	U(14)= 12 p=0.04	t(13)= -0.93 p=0.37	t(13)= -2.53 p=0.02	t(14)= -0.69 p=0.50	t(13)= -2.34 p=0.04	t(11)= -1.20 p=0.25	t(13)= -1.62 p=0.13	
**14 DPI**	t(13)= -3.07 p=0.009	t(13)= 1.98 p=0.07	t(13)= 0.64 p=0.53	t(13)= -0.29 p=0.77	t(13)= -0.11 p=0.91	t(13)= -0.01 p=0.99	t(13)= 1.41 p=0.18	
**30 DPI**	t(13)= 5.16 p<0.001	t(11)= -1.40 p=0.19	t(14)= -0.45 p=0.66	t(12)= -0.95 p=0.36	t(14)= -1.03 p=0.32	t(10)= 0.12 p=0.90	t(13)= -1.44 p=0.17	
**90 DPI**	U(13)= 12 P=0.04	t(13)= -0.93 p=0.37	t(13)= -2.53 p=0.02	t(14)= -0.69 p=0.50	t(13)= -2.34 p=0.04	U(13)= 18 p=0.28	t(13)= -1.90 p=0.08	
**150 DPI**	U(18)= 21 P=0.03	t(18)= -1.16 P=0.26	t(18)= -1.86 p=0.08	t(18)= -0.31 p=0.76	U(17)= 42 p=0.84	t(16)= 2.21 p=0.04	t(18)= -0.49 p=0.62	

Supplementary Table 1. In addition to microglial cell soma, perimeter changes can also be measured. Generally, differences in area and perimeter are mirrored, but occasionally only one is significantly different. Red *p* values indicate that the result is different from the area result (e.g., perimeter was insignificant while area was significant). "t" = student's t test; "U" = Wilcoxon rank sum (following failed normality).

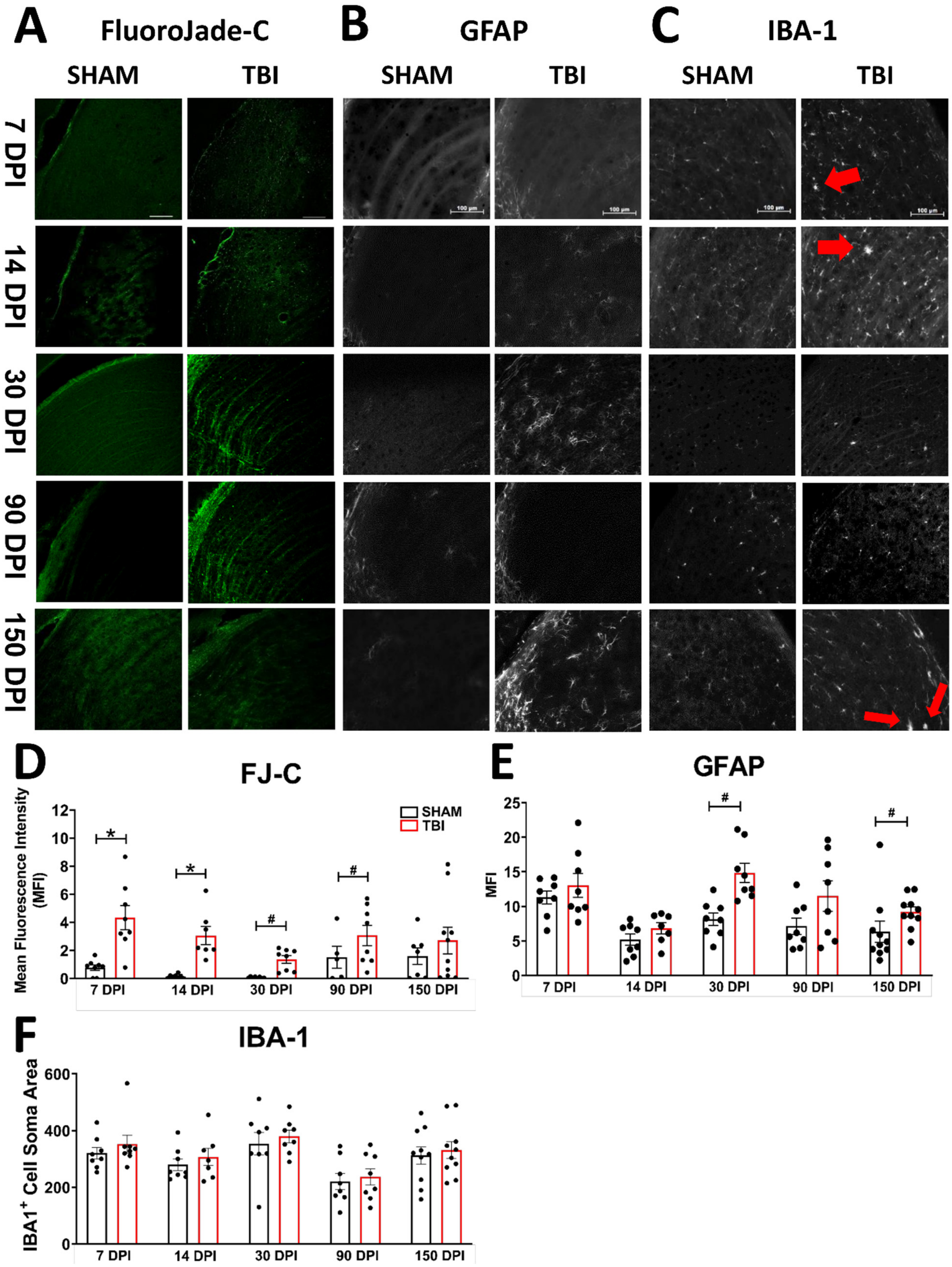

Figure S4. The LGN has a similar pattern of degeneration as the OT that persists throughout the time course.

Representative photomicrographs of dLGN (A) FJ-C staining, (B) GFAP stains, and (C) IBA-1 at all five time points examined. (D) As noted in the results, FJ-C MFI was significantly higher at all times except 150 DPI. (E) GFAP reactivity (MFI) was only significantly increased in TBI mice 30 and 150 DPI, with a delay in reactivity up to 30 DPI and (F) there were no significant increases in IBA-1 soma area. However, as you can see with the red arrows, a few ameboid-like microglia do show up in the dLGN at several time points examined. For FJ-C sham images, background was enhanced in order to visualize structures, and, for TBI images, contrast was enhanced to visualize staining more easily. Scale bars indicate 100μm and can be applied to all images in their respective stain. *p<0.001, # p<0.05.

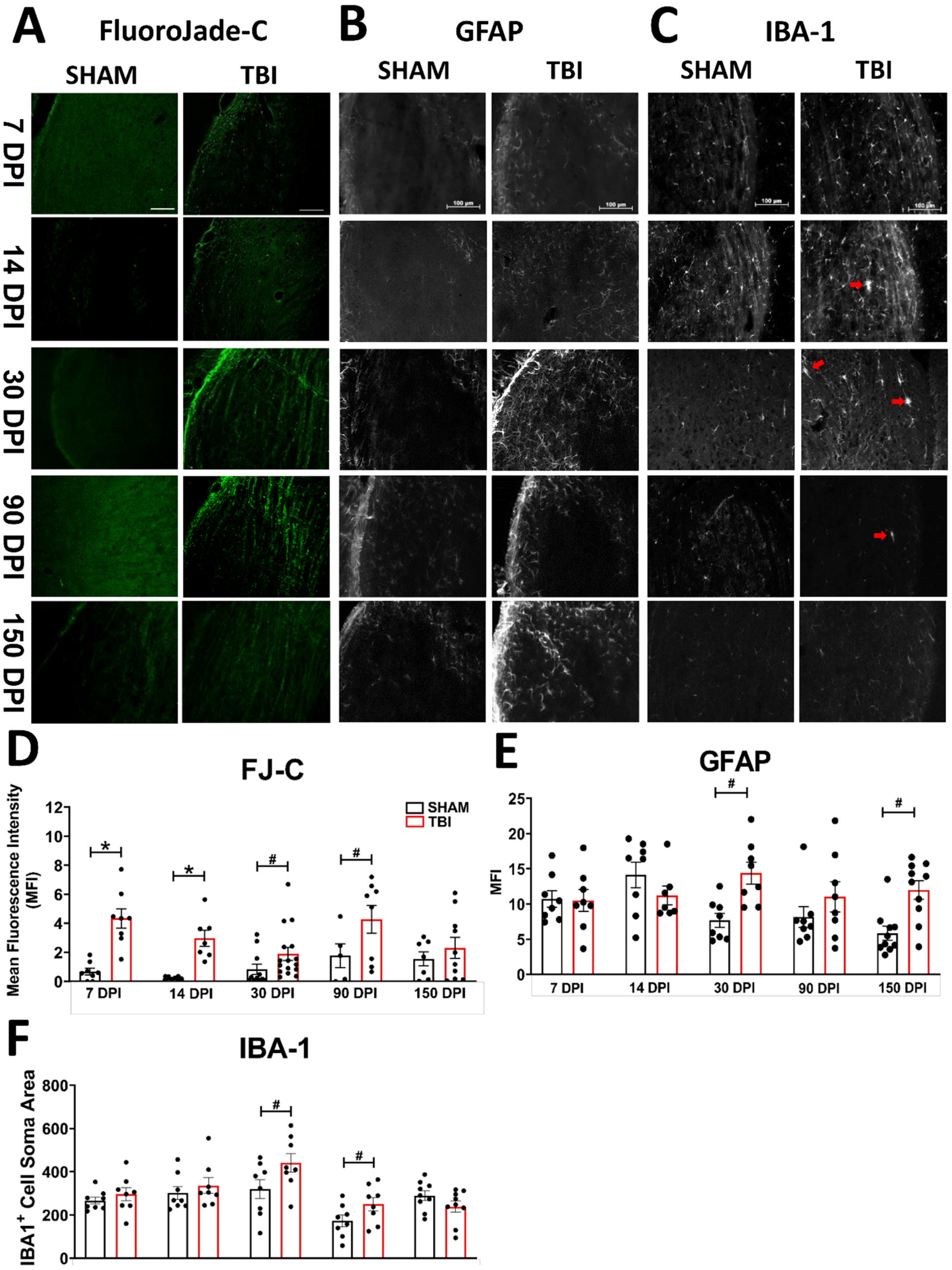

A
FluoroJade-C
SHAM
TBI
B
GFAP
SHAM
TBI
C
IBA-1
SHAM
TBI
7 DPI
14 DPI
30 DPI
90 DPI
150 DPI
D
FJ-C
Mean Fluorescence Intensity (MFI)
SHAM
TBI
E
GFAP
MFI
F
IBA-1
IBA1+ Cell Soma Area

Figure S5. From 30 DPI on, the dLGN shows two distinct populations of mice. 30, 90 and 150 DPI a bi-modal distribution arose within the TBI group for FJ-C staining intensity. (A) Low- and high-expression of FJ-C is significantly different; and, as in the OT, despite lower levels than high-FJ-C, the low-expressing group is still significantly higher than sham (B) as well as the high FJ-C expressing group (C). (D) 90 DPI there is also a bi-modal distribution where low and high groups are significantly different. However, at this time point, low expression is no longer different from sham (E) while high expressers continue to have significantly higher staining intensity (F). (G) Finally, the same results occur at 150 DPI, as in 90, with significant differences between expression groups within TBI, no differences between low FJ-C and sham (H), and high expression remains significantly higher than sham (I). (J) Representative 20x photomicrographs of low vs high expression of FJ-C in TBI mice at 30 DPI - contrast was enhanced to visualize staining more easily. Red bars indicate the high FJ-C expressing mice, and grey bars low FJ-C expression. These groups were distinguished by a median split. Scale bars represent 100 μm. MFI: Mean Fluorescence Intensity. *$p<0.05$, **$p<0.01$, ***$p<0.001$, ****$p<0.0001$

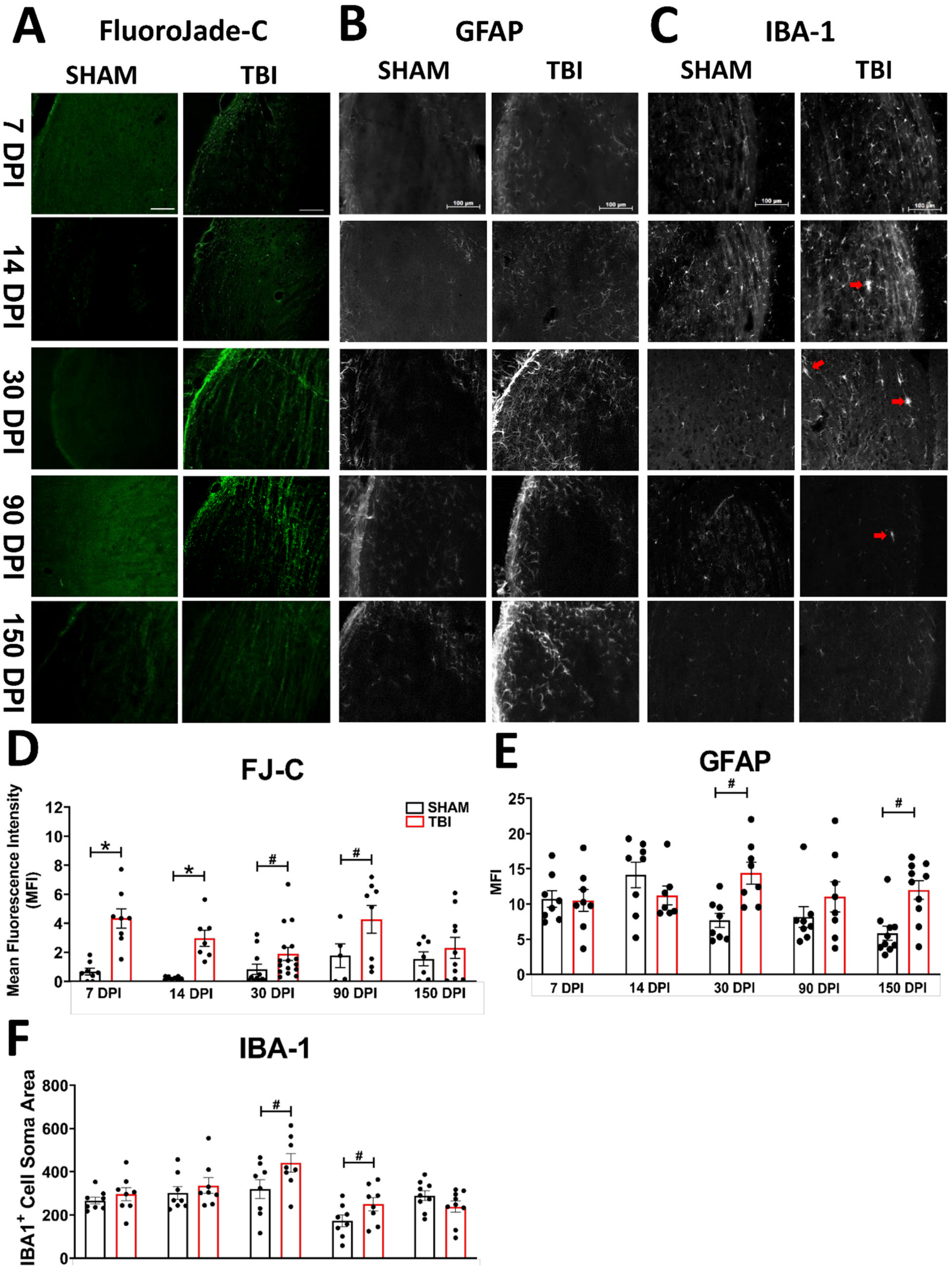

A
FluoroJade-C
B
GFAP
C
IBA-1
SHAM
TBI
7 DPI
14 DPI
30 DPI
90 DPI
150 DPI
D
FJ-C
Mean Fluorescence Intensity (MFI)
SHAM
TBI
E
GFAP
MFI
F
IBA-1
IBA1+ Cell Soma Area

Figure S6. The vLGN is affected by TAI in a similar manner as the dLGN save for delayed microglial activation. (A) Shows FJ-C representative 20x images for each time point**.** For Sham images, background was enhanced in order to visualize structures/tissue. For TBI images, contrast was enhanced to visualize punctate staining more easily. (D) There was significant degeneration in the vLGN until 150 DPI at which point little to no FJ-C was visible. (B) Shows representative 20x images for GFAP at each time point. (E) GFAP reactivity was only significantly elevated 30 and 150 DPI. (C) Shows representative 20x images of microglial morphology via IBA-1 staining at each time point. (F) Unlike the dorsal LGN, the vLGN's microglial response/phagocytic morphology is significantly increased at 30 and 90 DPI indicating that these regions, which contain different sets of projection neurons may respond differently to OT axon injury. Red arrows highlight amoeboid/larger soma microglial cells. Scale bars indicate 100μm and can be applied to all images in their respective stain. *$p<0.001$, # $p<0.05$.

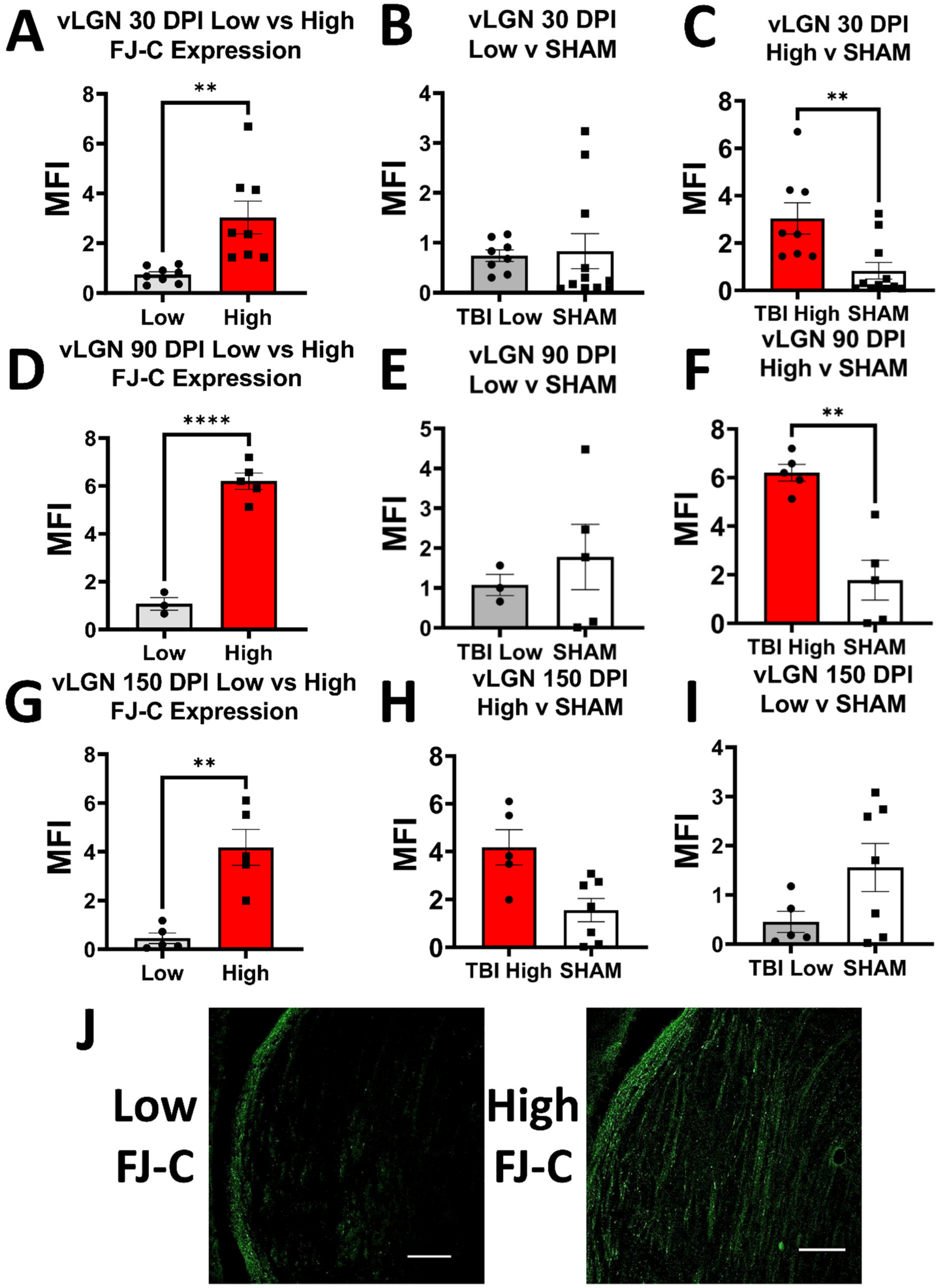
A
vLGN 30 DPI Low vs High
FJ-C Expression
B
vLGN 30 DPI
Low v SHAM
C
vLGN 30 DPI
High v SHAM
MFI
Low
High
TBI Low
SHAM
TBI High
D
vLGN 90 DPI Low vs High
FJ-C Expression
E
vLGN 90 DPI
Low v SHAM
F
vLGN 90 DPI
High v SHAM
G
vLGN 150 DPI Low vs High
FJ-C Expression
H
vLGN 150 DPI
High v SHAM
I
vLGN 150 DPI
Low v SHAM
J
Low
FJ-C
High
FJ-C

Figure S7. From 30 DPI degeneration in the vLGN shows two distinct populations of mice. (A–C) Show results of low vs high FJ-C expression comparisons against each other and sham at 30 DPI at which low FJ-C expressing mice are not different than sham FJ-C expression. (D–F) Show results for low vs sham FJ-C expression at 90 DPI where low expression is still not different from sham. (G-I) While low vs high expression within the TBI group is significantly different, neither group is different from sham at 150 DPI. (J) Representative 20x photomicrographs of 30 DPI FJ-C comparing high and low TBI FJ-C expression. Contrast was enhanced in order to visualize FJ-C positive staining. Scale bars indicate 100μm. See other figures for sham comparison. *p<0.05, **p<0.01, ***p<0.001, ****p<0.0001

Table S2. dLGN vs vLGN FJ-C ANOVAs (No effect of location within LGN)

Time Point	N dLGN		N vLGN		F statistic		*p* value
7 DPI	SHAM	iTON	SHAM	iTON	Injury	F (1,31) = 24.38	< 0.001
	8	7	8	8	Location	F (1,31) = 0.02	0.89
					Interaction	F (1,31) = 0.02	0.88
14 DPI	SHAM	iTON	SHAM	iTON	Injury	F (1,29) = 35.75	< 0.001
					Location	F (1,29) = 0.0006	0.98
	7	8	7	8	Interaction	F (1,29) = 0.06	0.80
30 DPI	SHAM	iTON	SHAM	iTON	Injury	F (1,38) = 8.15	0.008
					Location	F (1,38) = 3.25	0.10
	8	11	8	11	Interaction	F (1,38) = 10.68	0.008
90 DPI	SHAM	iTON	SHAM	iTON	Injury	F (1,25) = 2.89	0.12
					Location	F (1,25) = 3.28	0.09
	5	8	5	8	Interaction	F (1,25) = 1.34	0.27
150 DPI	SHAM	iTON	SHAM	iTON	Injury	F (1,33) = 0.75	0.40
					Location	F (1,33) = 0.86	0.37
	7	10	7	10	Interaction	F (1,33) = 0.67	0.43

The dorsal and ventral LGN show similar levels of degeneration after TAI in the OT. Statistics for dLGN vs vLGN comparison within FluoroJade-C staining are given. 2-way ANOVA revealed no effect of location and no interactions (p > 0.05), but an effect of injury was still true for each time point except 150 DPI.

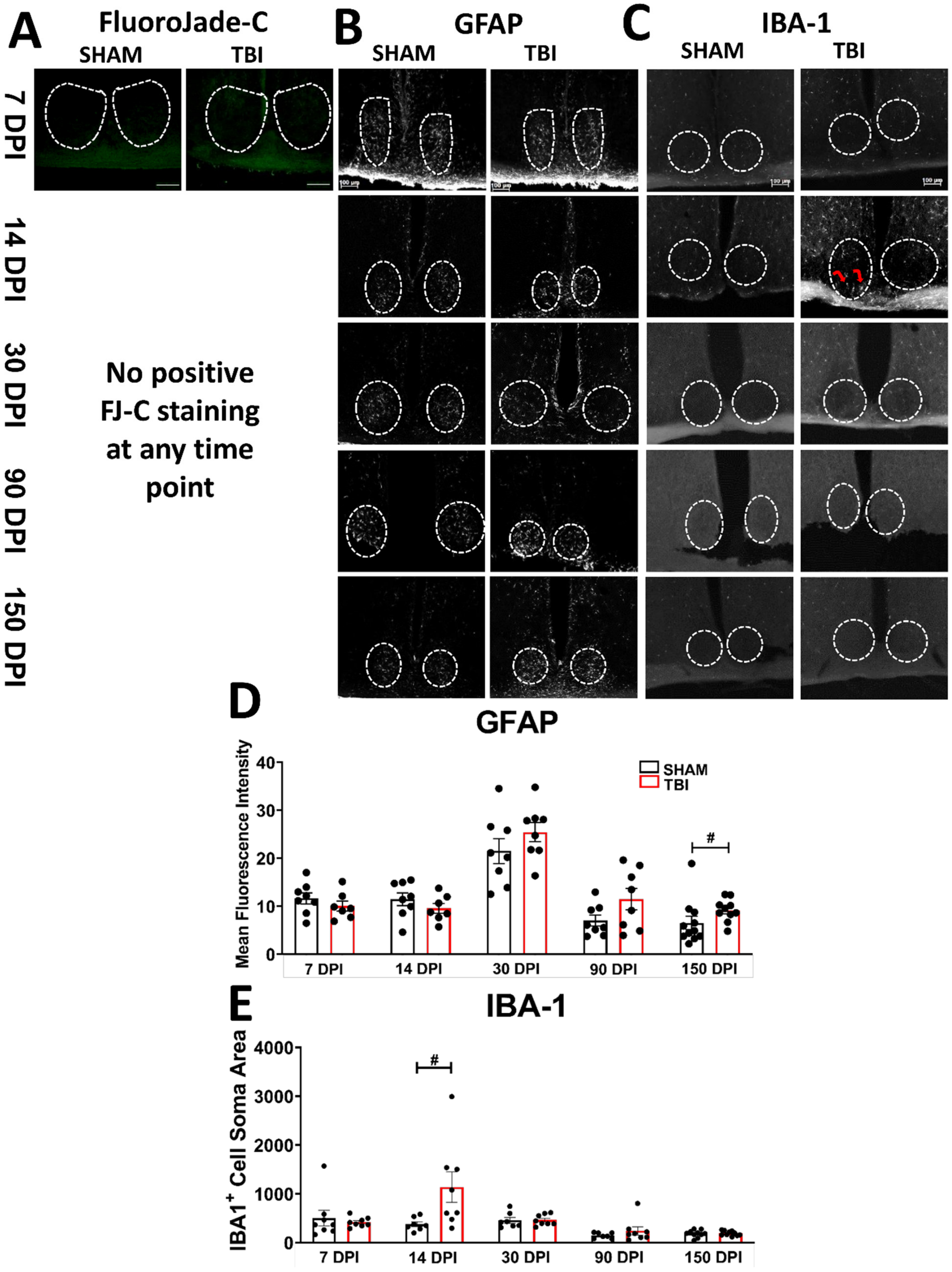

A
FluoroJade-C
SHAM
TBI
B
GFAP
SHAM
TBI
C
IBA-1
SHAM
TBI
7 DPI
14 DPI
30 DPI
90 DPI
150 DPI
No positive FJ-C staining at any time point
D
GFAP
Mean Fluorescence Intensity
SHAM
TBI
#
7 DPI
14 DPI
30 DPI
90 DPI
150 DPI
E
IBA-1
IBA1+ Cell Soma Area
#
7 DPI
14 DPI
30 DPI
90 DPI
150 DPI

Figure S8. Despite a general lack of cellular disturbance to TAI in the SCN, microglia alone become reactive only at 14 DPI. (A) Shows a representative 20x image of FJ-C staining at 7 DPI in the SCN where we saw no positive staining at any time. (B) Depicts 20x photomicrographs of GFAP staining for which (D) there was no significant change at any time. (C) Shows 20x images of IBA-1 staining at which (E) there were significantly increased somata found at 14 DPI. White dashed circles show the area examined, red arrows pinpoint examples of larger microglia in injured mice. Scale bars indicates 100μm and can be applied to all images in their respective stain. *p<0.001, # p<0.05.

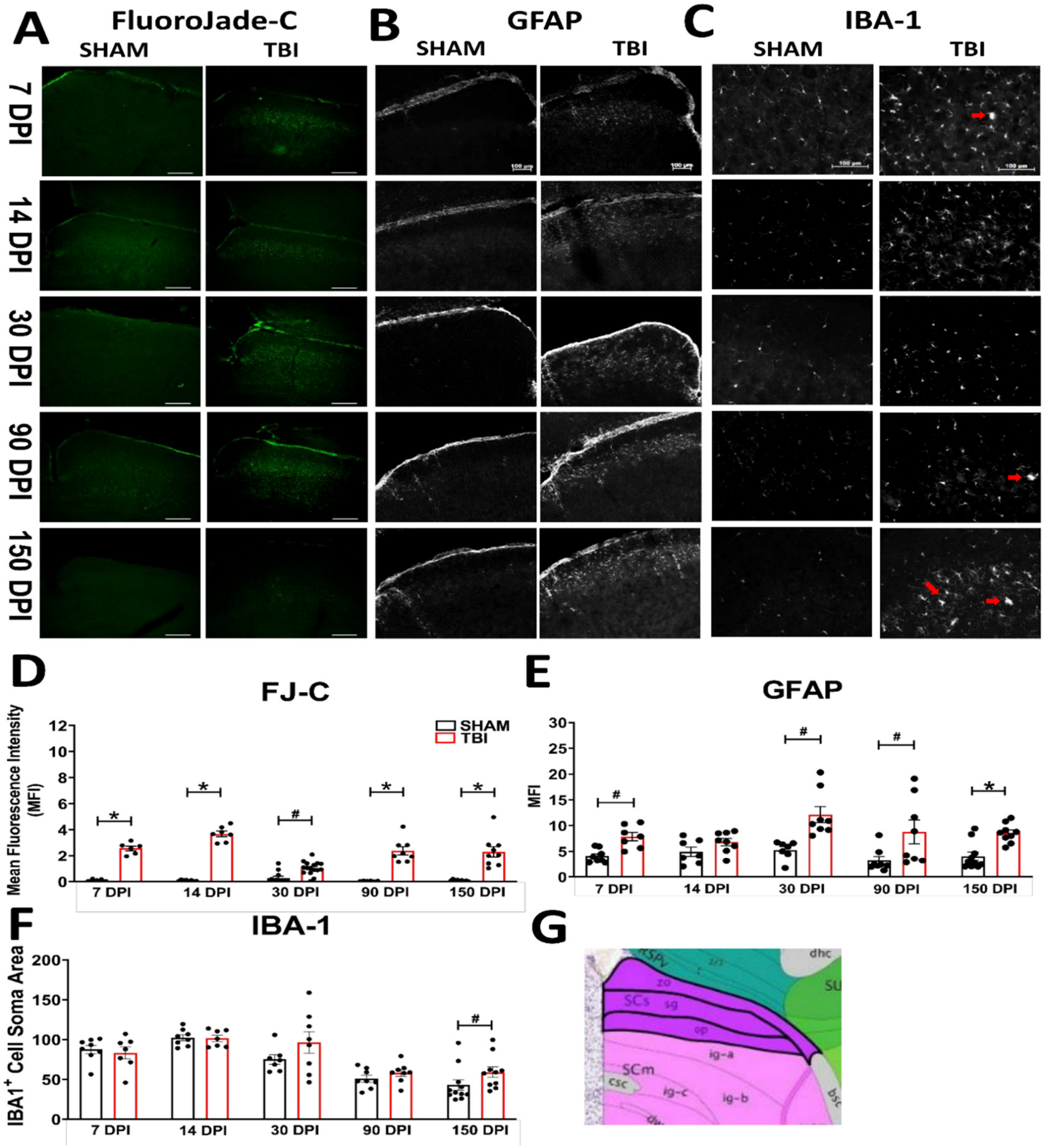

Figure S9. The input layers of the superior colliculi are not spared from degeneration or glial reactivity after TON, but inflammatory activation is chronically delayed. Representative 20x photomicrographs of SC FJ-C staining (A) GFAP staining (B) and IBA-1 staining (C) for each time point examined are shown. (D) There was significantly elevated fluorescence of FJ-C limited to the

superficial input layers of the SC at all times. (E) Astrocyte increased reactivity was also significantly elevated, but further showed a potential for recovery at 14 DPI. (F) Microglial response to injury, measured as increased soma size to indicate the presence of phagocytic behavior was not significantly changes until 150 DPI implicating them as responders to chronic cellular changes after TAI. (G) Allan brain atlas image used to show layers of SC – zonal, superficial grey, and optic zone – in bold black and dark purple (C). Overexposure at the edge of the BoSC or SC was not included in analyses Additional images to show splitting of FJ-C groups are provided in sup. fig. 10. Scale bars indicate 100 μm. *p<0.001, # p<0.05. (G) Image credit: Allen Institute found in reference to the Mouse, P56, Coronal Atlas (http://atlas.brainmap.org/atlas?atlas=1#atlas=1&plate=100960053&structure=549&x=5286.000061035156&y=3750&zoom=-3&resolution=10.47&z=7).

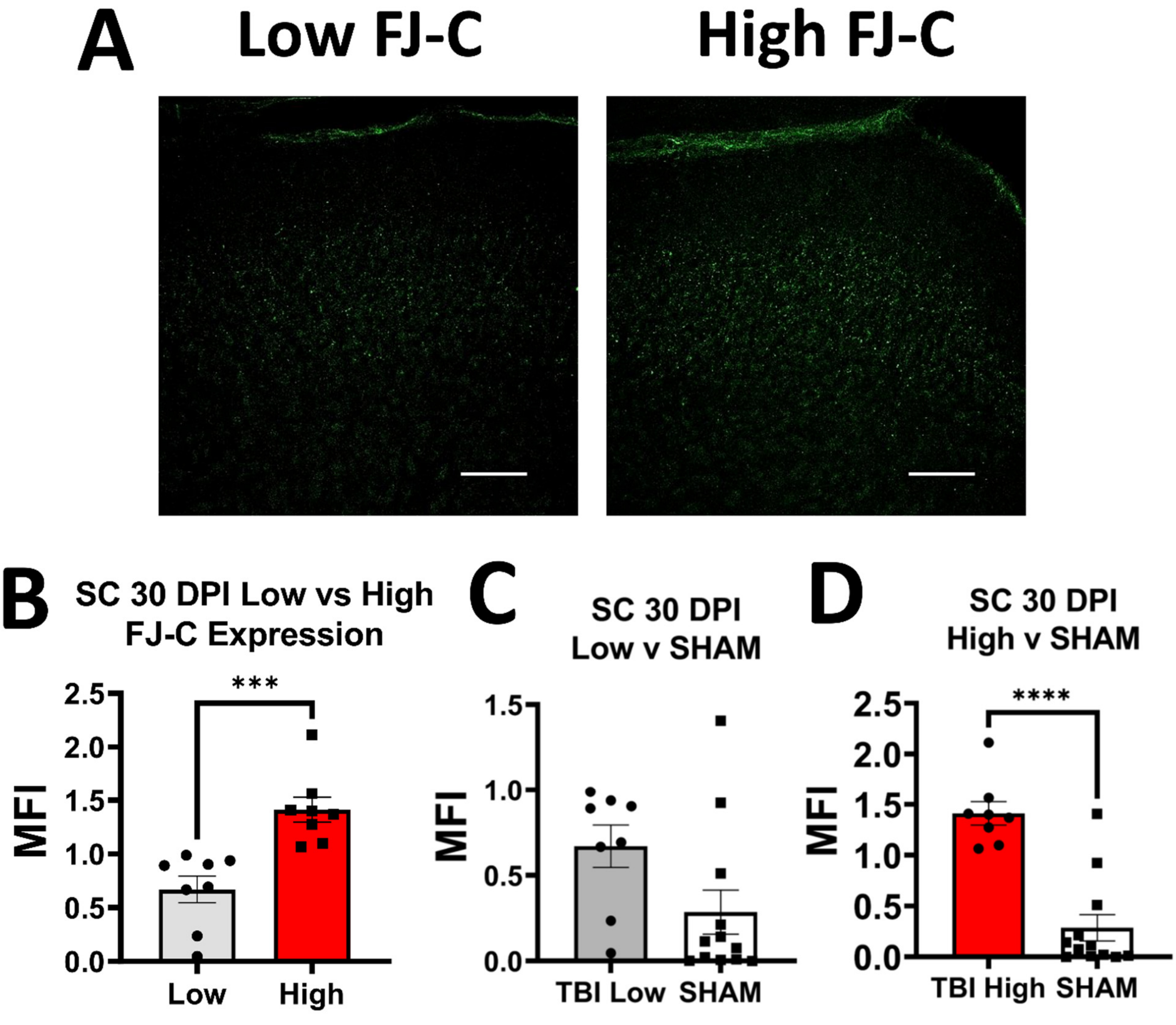

Figure S10. At 30 DPI degeneration in the SC shows two distinct populations of mice with a low group showing potential resiliency to injury. Visually, splitting of the TBI group was evident in superficial layers of the SC. (A) Shows representative images (20x) of the low and high SC TBI mice at 30 DPI. (B) 30 DPI the two groups were split at their median with low (grey bars) vs high (red bars) FJ-C expression which were significantly different from each other. (C) The low group was not significantly different from sham while the high group (D) remained significantly worse. Contrast was enhanced to visualize staining more easily. Scale bars indicate 100 μm. *p<0.05, **p<0.01, ***p<0.001, ****p<0.0001

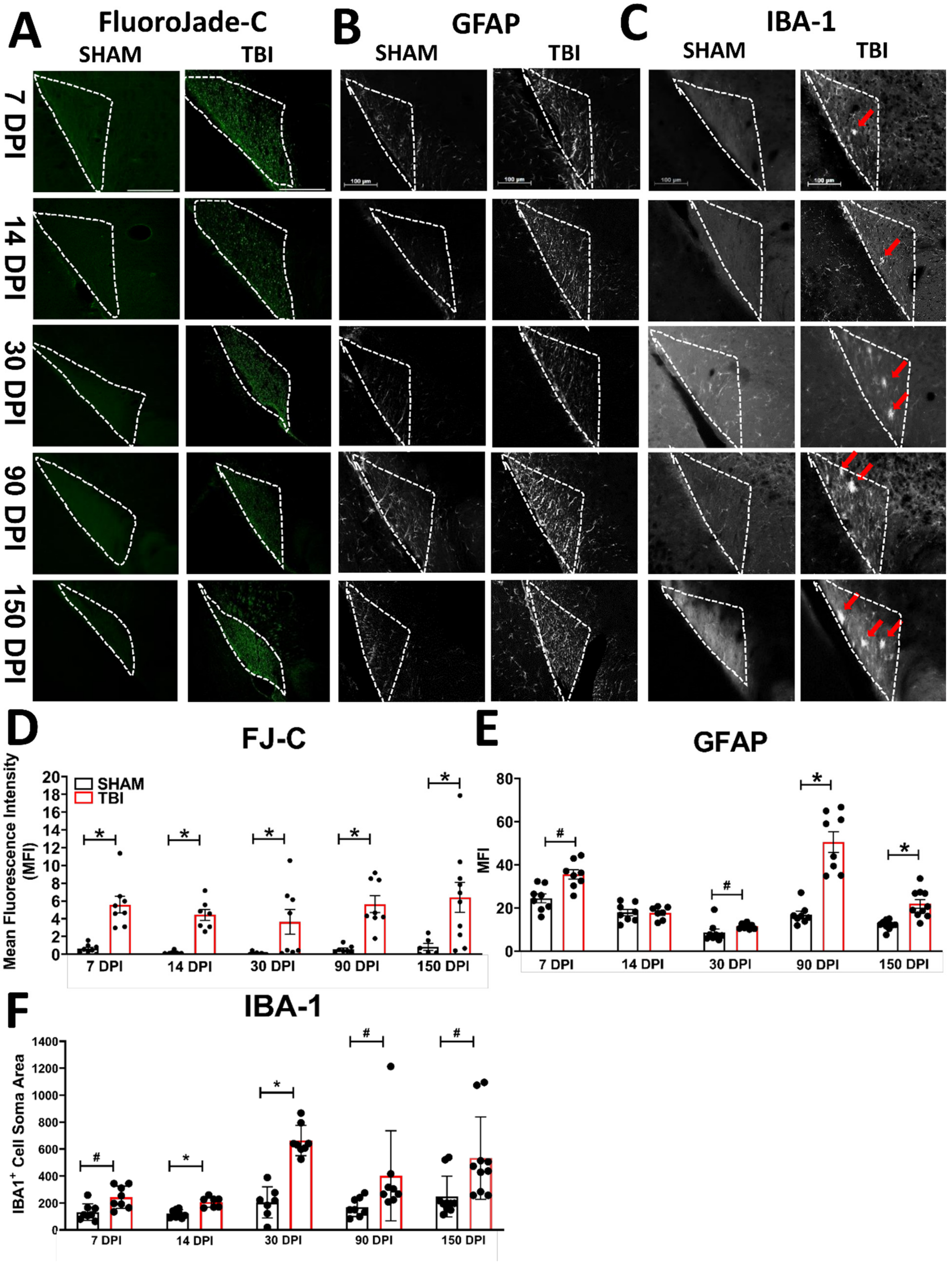
A
FluoroJade-C
SHAM
TBI
B
GFAP
SHAM
TBI
C
IBA-1
SHAM
TBI
7 DPI
14 DPI
30 DPI
90 DPI
150 DPI
D
FJ-C
Mean Fluorescence Intensity (MFI)
SHAM
TBI
7 DPI
14 DPI
30 DPI
90 DPI
150 DPI
E
GFAP
MFI
7 DPI
14 DPI
30 DPI
90 DPI
150 DPI
F
IBA-1
IBA1⁺ Cell Soma Area
7 DPI
14 DPI
30 DPI
90 DPI
150 DPI

Figure S11. The white matter tracts of the accessory optic system are also affected by OT TAI chronically. (A) Representative images of FJ-C staining for each time point in the Dorsal Terminal Nuclei of sham and TBI mice 7, 14, 30, 90 and 150 DPI (40x magnification). (B) Shows GFAP 40x images in the DTN for Sham vs TBI 7, 14, 20, 90 and 150 DPI. (C) Depicts 40x images of IBA-1 stained DTN for all times. Red arrows pinpoint larger microglia. White dotted lines surround the region analyzed. (D) FJ-C expression was present throughout the time course. (E) GFAP immunoreactivity was also elevated chronically but may also indicate a critical period at 14 DPI. (F) IBA-1 soma size was significantly increased throughout the time course. Scale bars indicate 100μm. *p<0.001, # p<0.05.

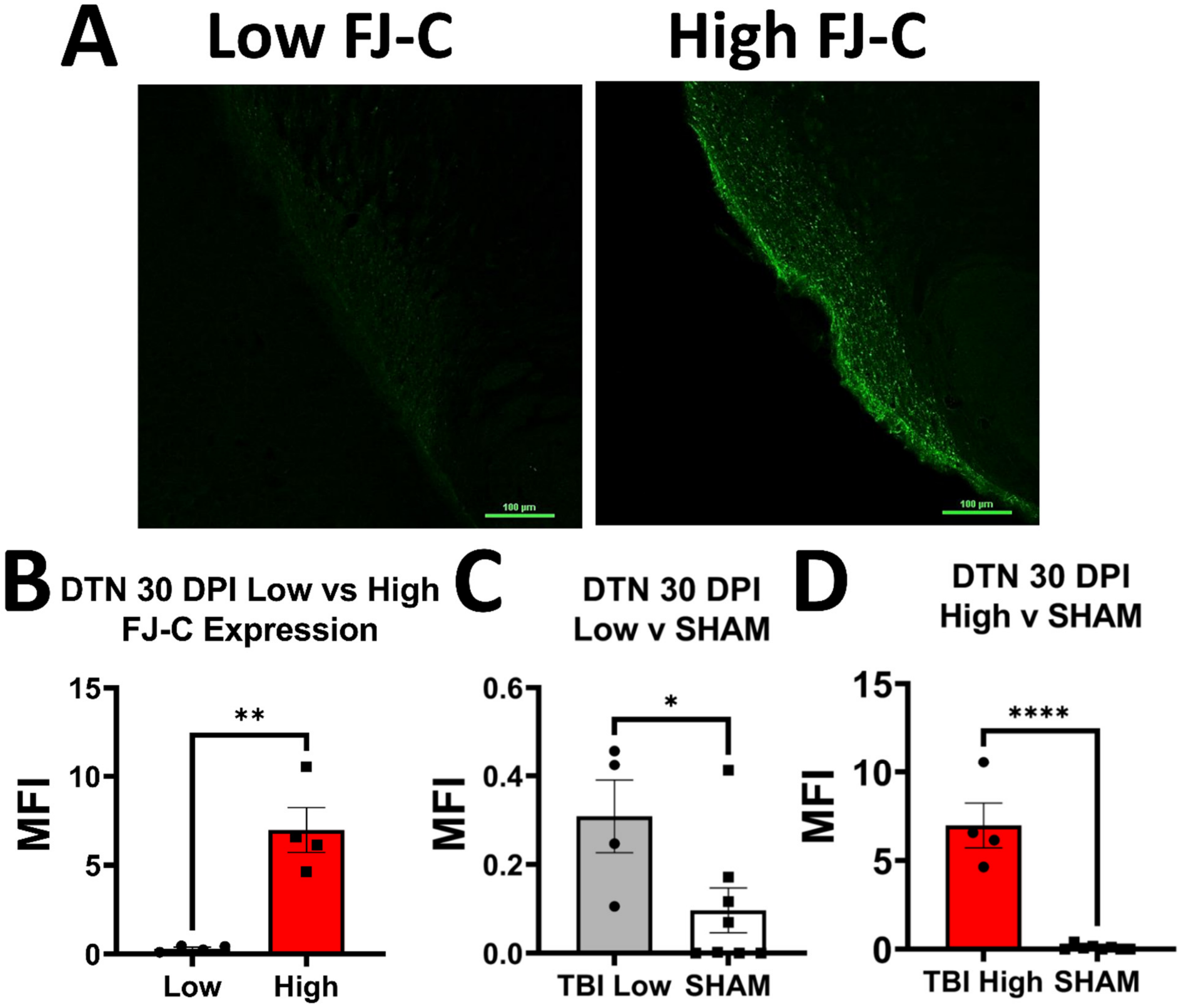

Figure S12. At 30 DPI, mice continue to show bi-modal distribution of FJ-C reactivity in the DTN of the accessory optic system. (A) Shows representative 40x images from 30 DPI TBI mice. Contrast was enhanced to visualize staining more easily. (B) Shows the division of the low vs high FJ-C expressing groups, which were significantly different from each other. (C) Low FJ expressing TBI mice no longer being different from sham, while (D) high FJ-C expressers remain significantly higher than sham. Scale bars indicate 100μm. *p<0.05, **p<0.01, ***p<0.001, ****p<0.0001

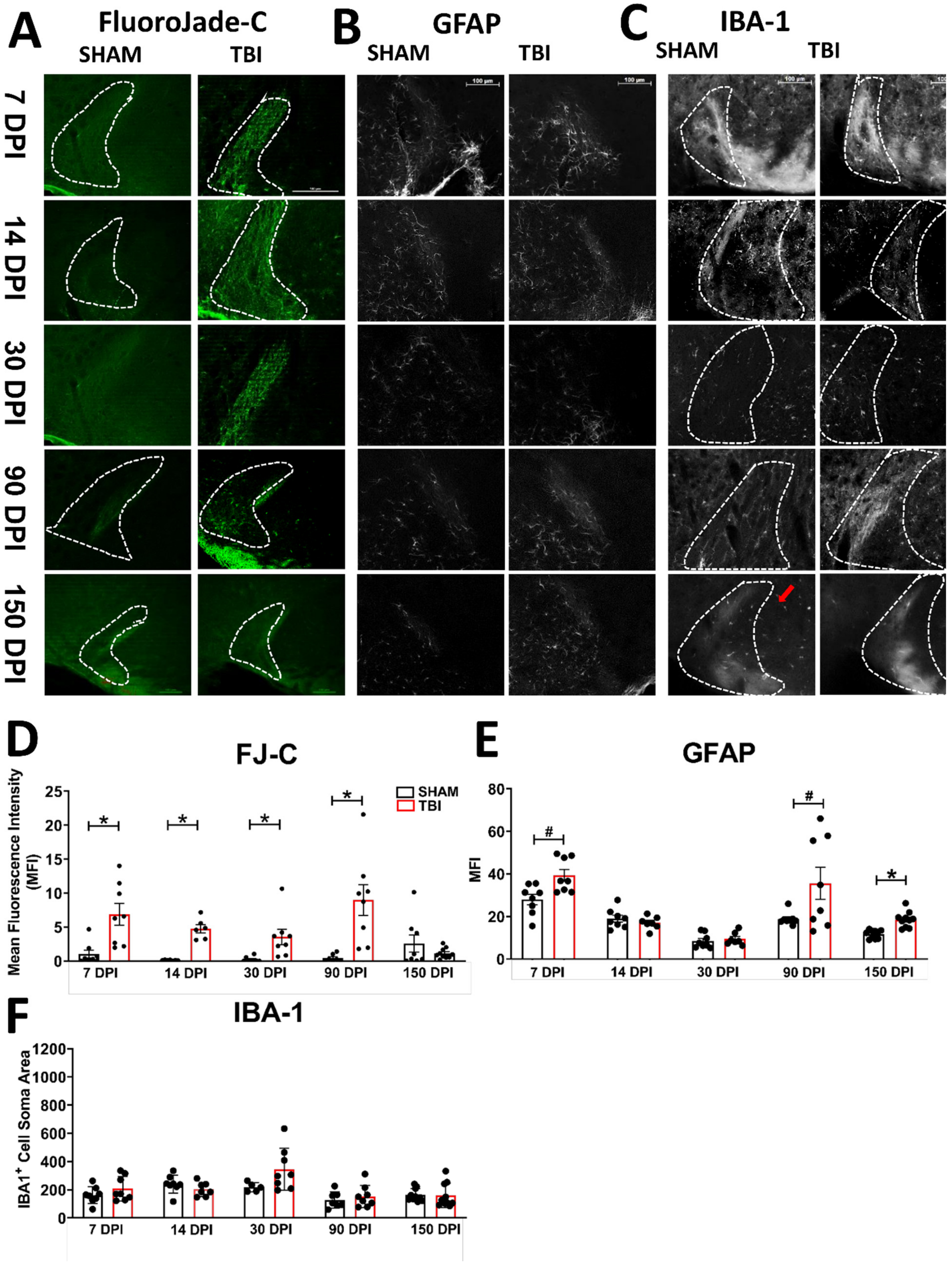
A
FluoroJade-C
SHAM
TBI
B
GFAP
C
IBA-1
7 DPI
14 DPI
30 DPI
90 DPI
150 DPI
D
FJ-C
Mean Fluorescence Intensity (MFI)
SHAM
TBI
E
GFAP
MFI
F
IBA-1
IBA1+ Cell Soma Area

Figure S13. The MTN of the accessory optic system shows nearly identical response to TAI as the DTN. Representative Images of each time point for (A) FJ-C, (B) GFAP, and (C) IBA-1 expression in the MTN. (D) The MTN had significantly elevated intensity of FJ-C at all times except 150 DPI. (E) As in other regions, astrocyte response is significantly elevated early (7 DPI) with subacute recovery (14 and 30 DPI) followed by chronic resurgence (190 and 150 DPI). (F) There were no microglial changes in the MTN. Scale bars indicate 100μm. *p<0.001, # p<0.05.

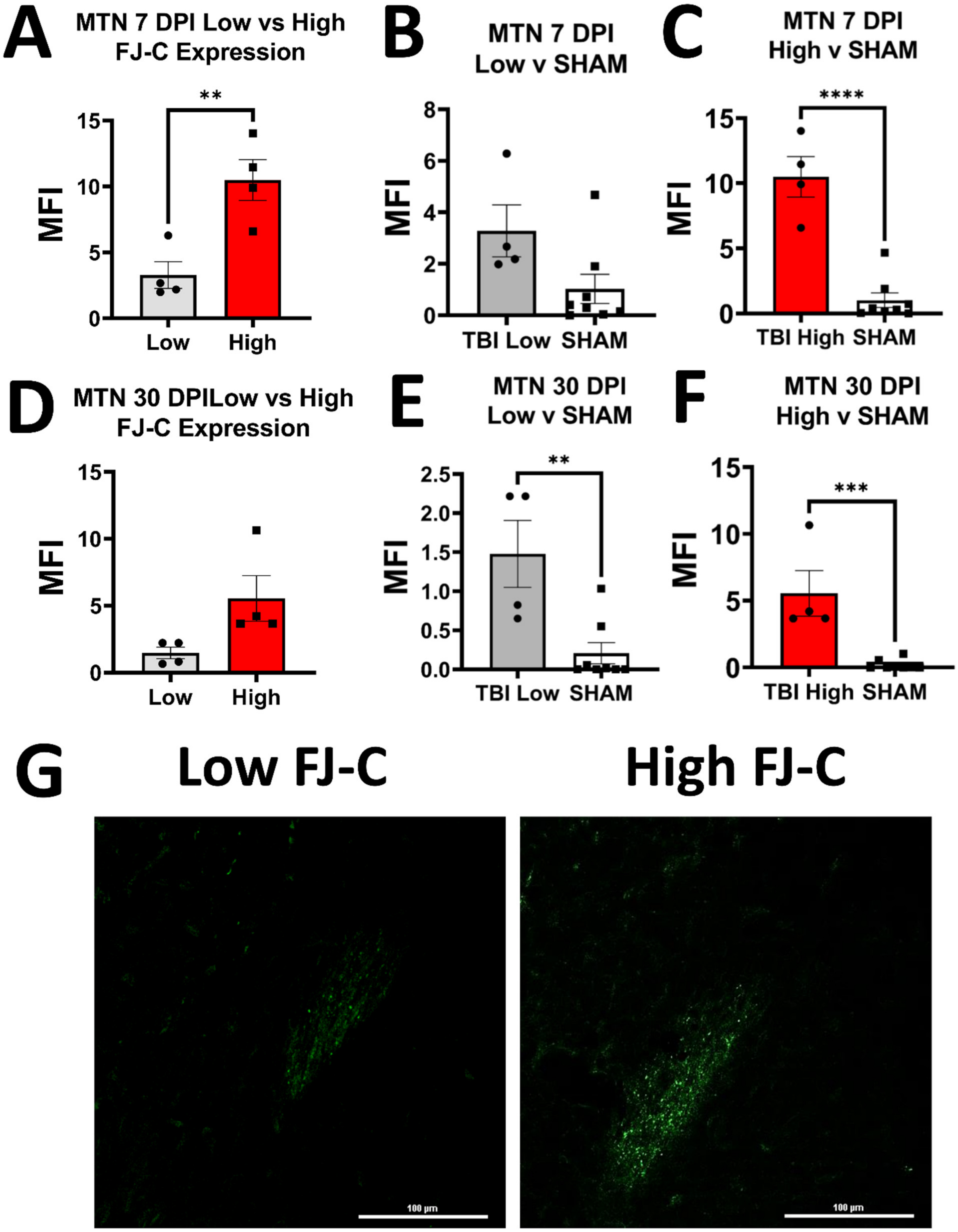

Figure S14. The MTN shows the potential for a group of resilient mice acutely and sub acutely. (A) Unlike other regions, the MTN shows a bi-modal distribution as early as 7 DPI with two significantly different populations of high and low- expression of FJ-C within the TBI group. (B) The low FJ-C expressing mice have low to no degeneration,

similar to sham, (C) but their higher TBI counterparts remain significantly higher than sham. This distribution is also present 30 DPI, (D) but the groups are not different from each other and both (E) low and (F) high expressers present with significantly higher FJ-C presence than sham. (G) Representative 40x images of 7 DPI low and high FJ-C. Contrast was enhanced to visualize staining more easily. Scale bars indicate 100μm. *p<0.05, **p<0.01, ***p<0.001, ****p<0.0001

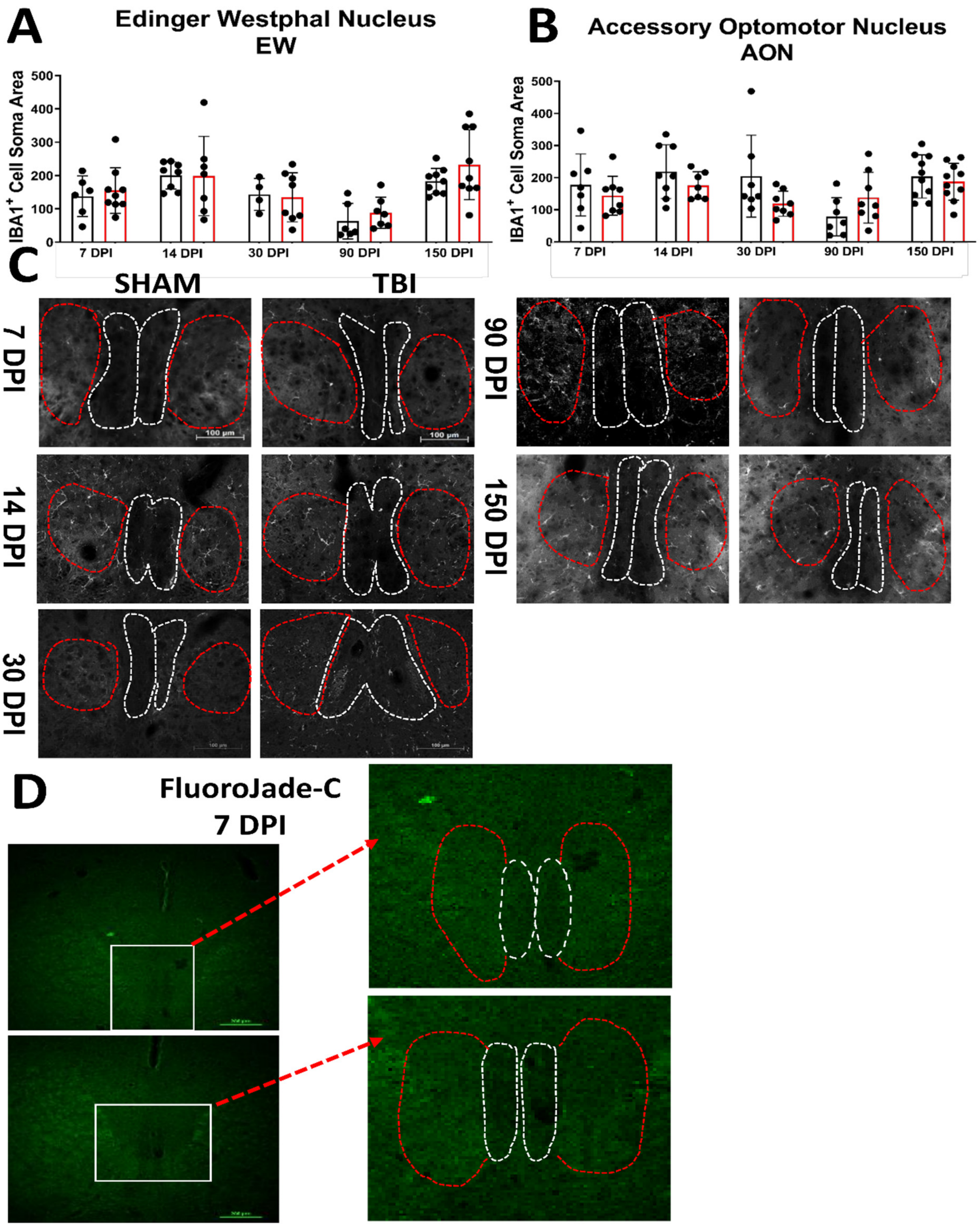

Figure S15. Pupillary control regions are not affected by TAI. (A) The Edinger- Westphal nuclei show no enlargement of IBA-1 cells at any time point, (C) nor does the nearby AON. (B) Shows representative 20x photomicrographs of these regions at each time point – red dotted regions indicate the AON, white dotted regions outline the EW. (D) Shows a lack of FJ-C in these regions, which was true of every time-point examined. Left images were taken at 40x with zoom from the white box following the red arrow to the right. Not shown, there was also no positive GFAP staining in these regions. Scale bars indicate 100µm.

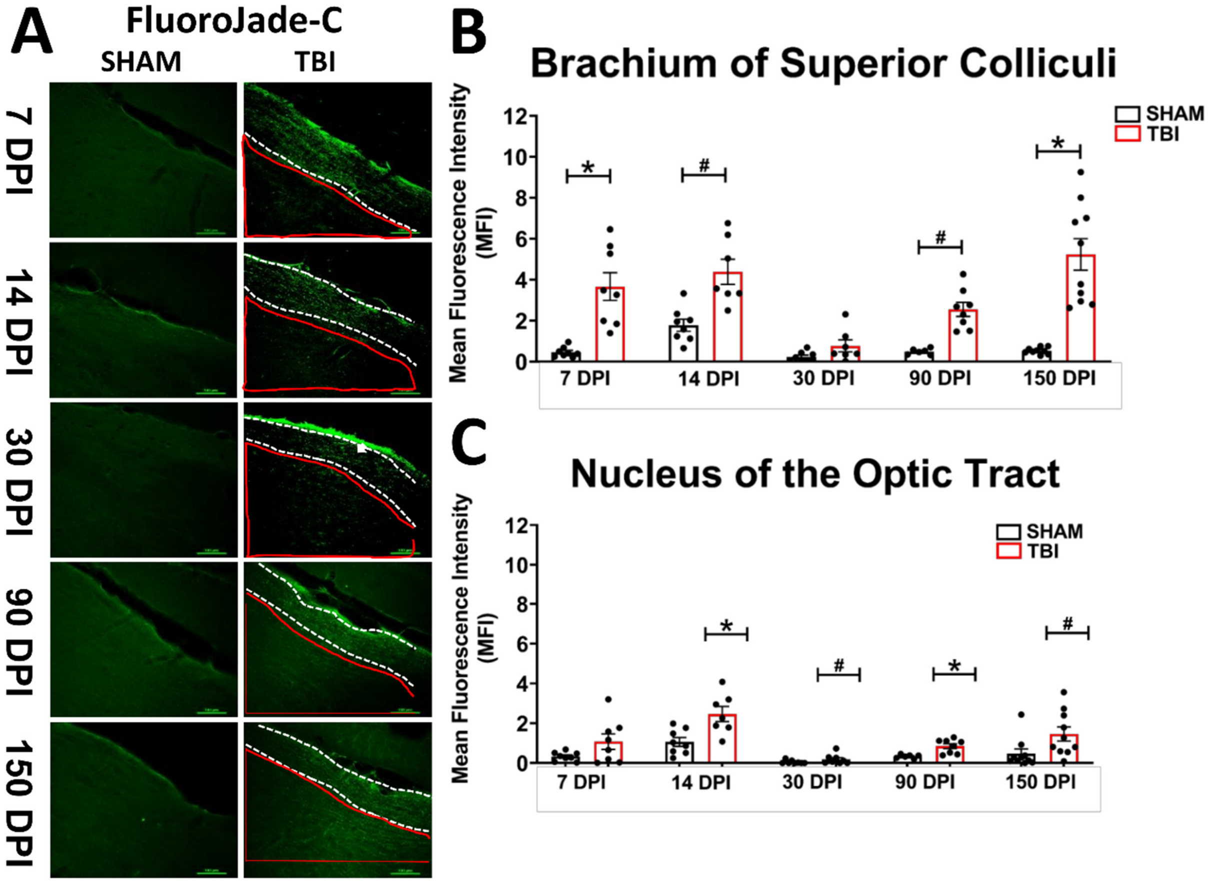

Figure S16. The BoSC and secondary OT projection site – the NOT – also contain degenerating axons chronically after TAI. (A) 20x Images of FJ-C 7,14, 30, 90, and 150 DPI sham vs TBI. (B) There is significantly increased degeneration in the BoSC at all times except the critical 30 DPI, which we see in other regions. (C) It takes up to 14 DPI for degeneration to become present in the NOT after which it remains until 150 DPI. White dotted line indicates BoSC and red line outlines NOT. Scale bars indicate 100µm. *$p<0.001$, # $p<0.05$.

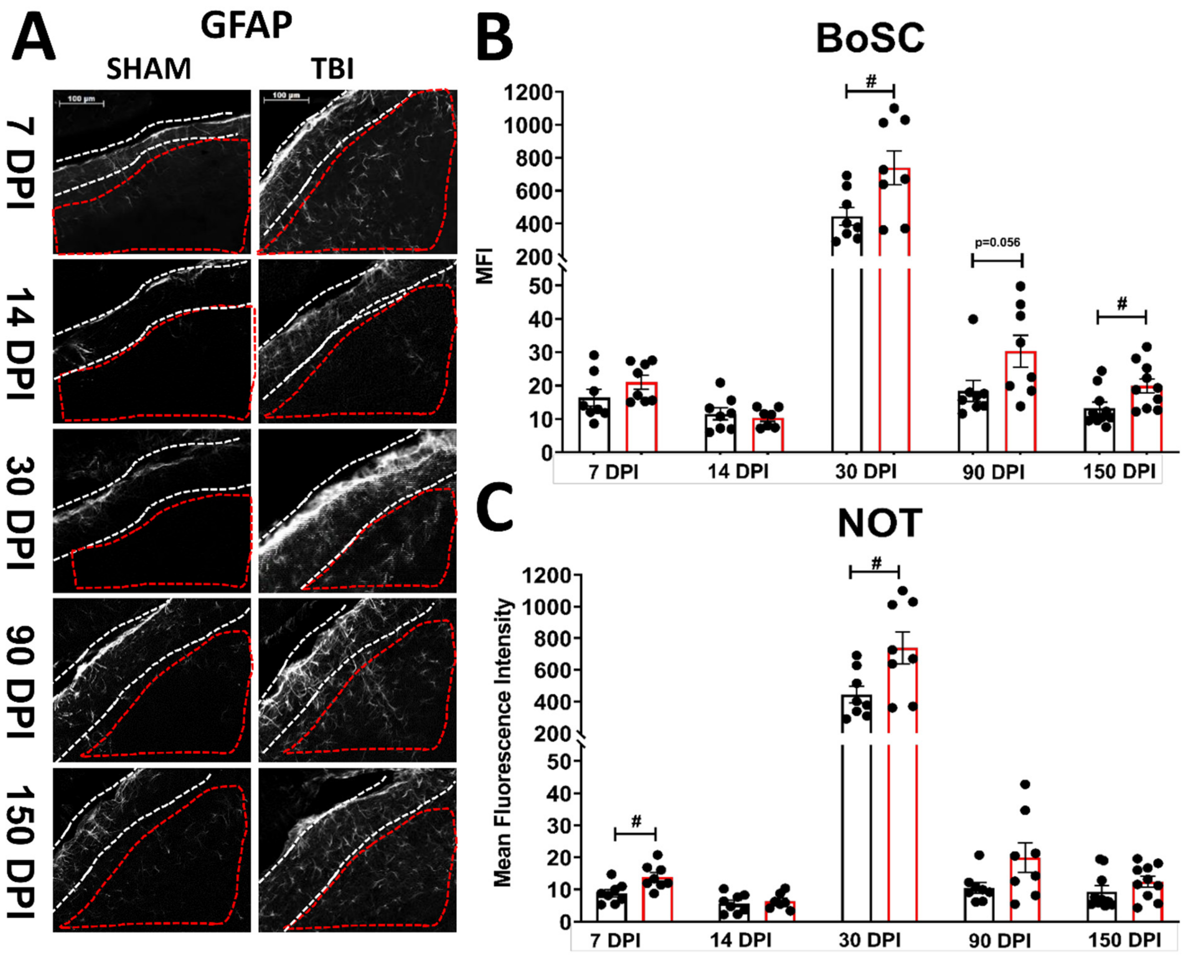

Figure S17. Astrocyte reactivity is immediate in the NOT and delayed in the BoSC. (A) Shows GFAP staining images of the BoSC (white lines) and NOT (red outline) at each time point. (B) The BoSC is a white matter branch of the OT and contains some output axons of the SC the project to the NOT does not show increased astrocyte GFAP intensity until 30 DPI after which it remains up to 150 DPI. (C) In the NOT, this glial reactivity pattern is more reminiscent of direct OT projection regions with early increases, recovery at 14 DPI, and resurgence 30 DPI. Because each time point represents a different set of mice, when the replacement 30 DPI cohort was stained, the fluorescence was higher, so B and C have a break in the y axis between 50 and 200. Scale bars indicate 100 µm. # $p<0.05$.

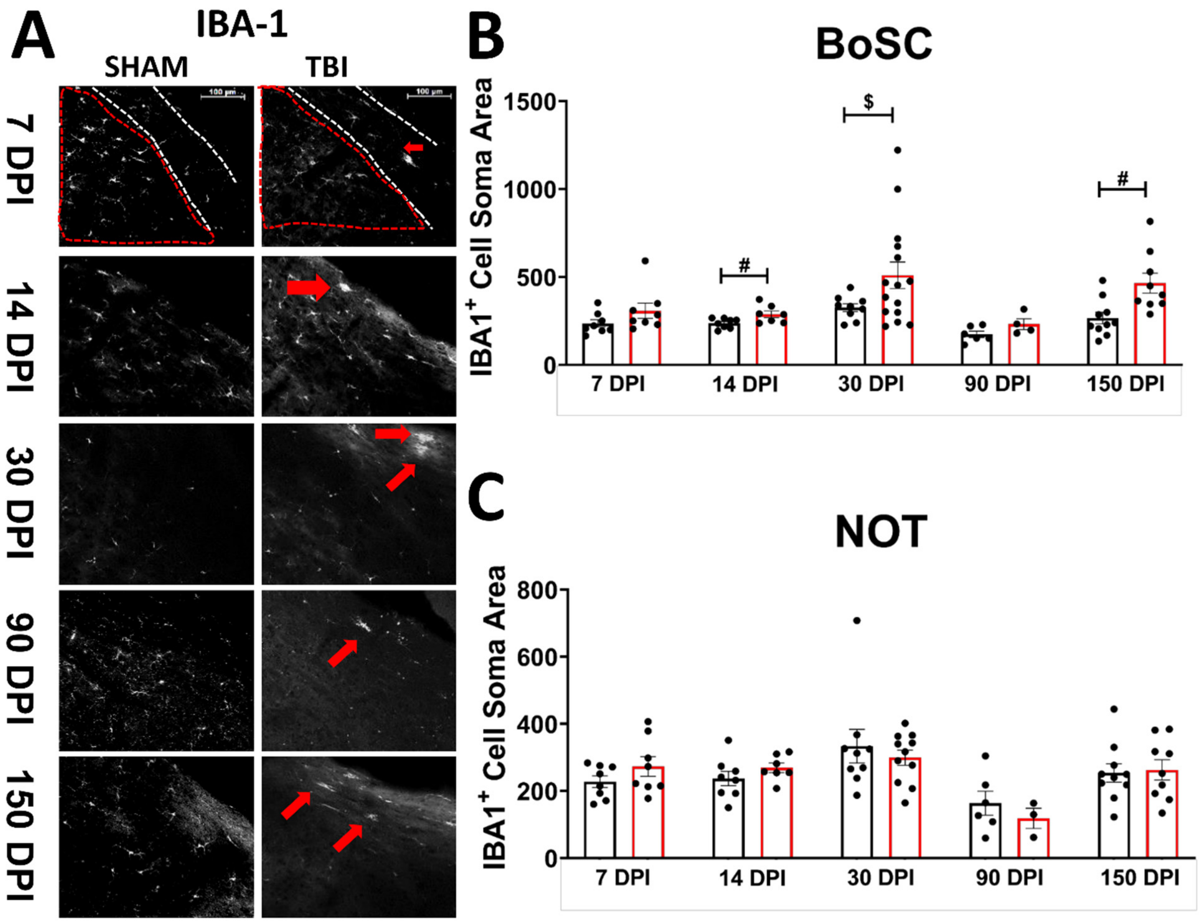

Figure S18. Increased microglial somata are only present in the white-matter BoSC and not the NOT. (A) Shows representative images for each time point of the BoSC and NOT for IBA-1 staining. Red arrows show increased microglia, white outlines indicate BoSC, and red outlines mark the NOT. (B) Microglial soma size is significantly larger in the BoSC 14, 30, and 150 DPI. (C) There is no change to microglia in the NOT at any time point examined. Scale bars indicate 100 μm. # $p<0.05$, $ $p<0.05$ for iba-1 perimeter reported in supplementary table 2.

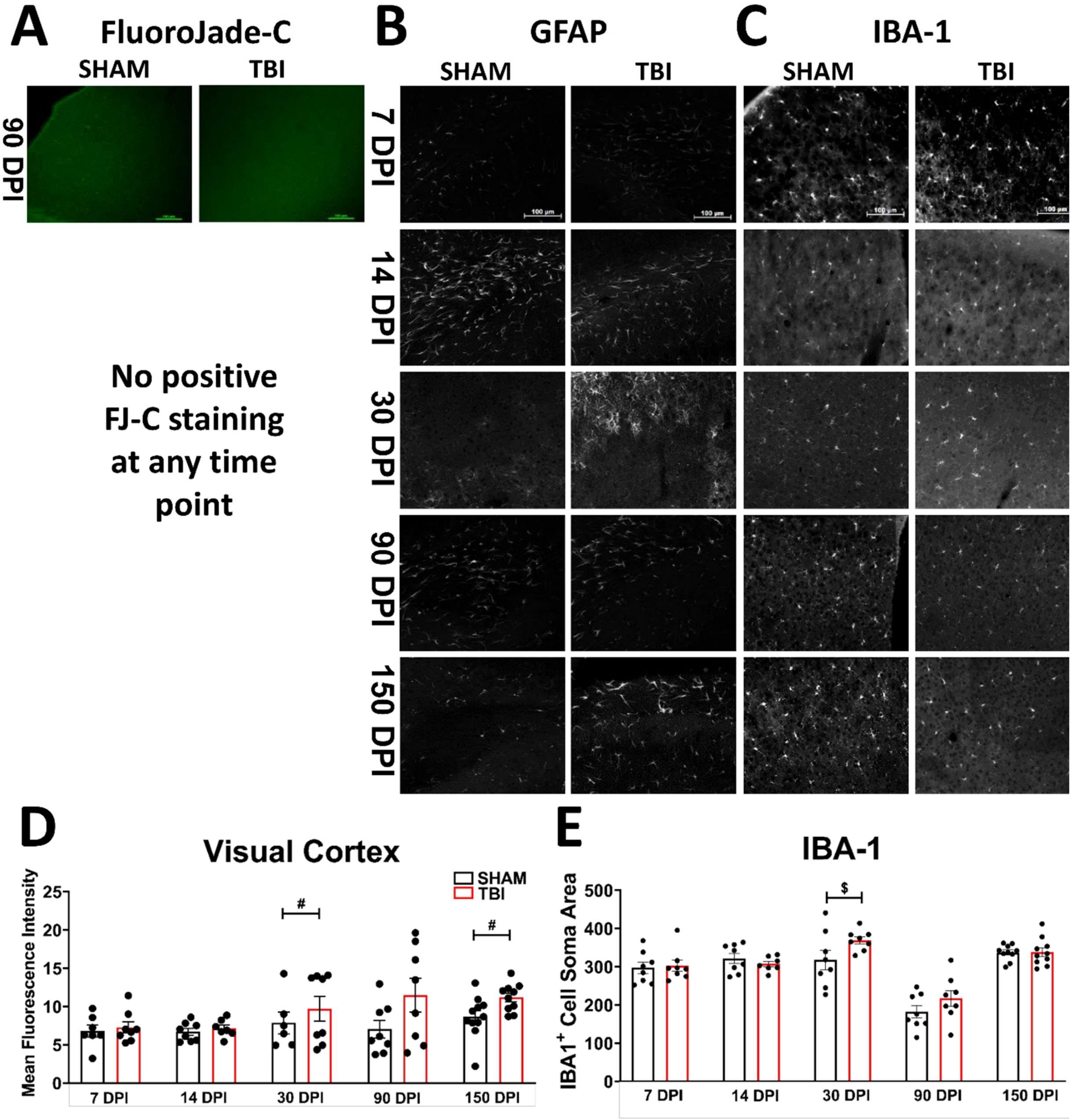

Figure S19. Despite a lack of degeneration, glial cells show increased response to injury in the visual cortex by 30 DPI. (A) Shows a representative 20x image of FJ-C staining at 90 DPI in the VC where we saw no positive staining at any time. (B) Depicts 20x photomicrographs of GFAP staining for which there were significant changes not only to reactivity at 30 DPI and 150 DPI (D), but also astrocyte morphology (see the B 30 DPI images). (C) Shows 20x images of IBA-1 staining at which (E) there were significantly increased somata perimeter found at 30 DPI. Scale bars indicate 100 μm. # p<0.05, $ p<0.05 for iba-1 perimeter reported in supplementary table 2.

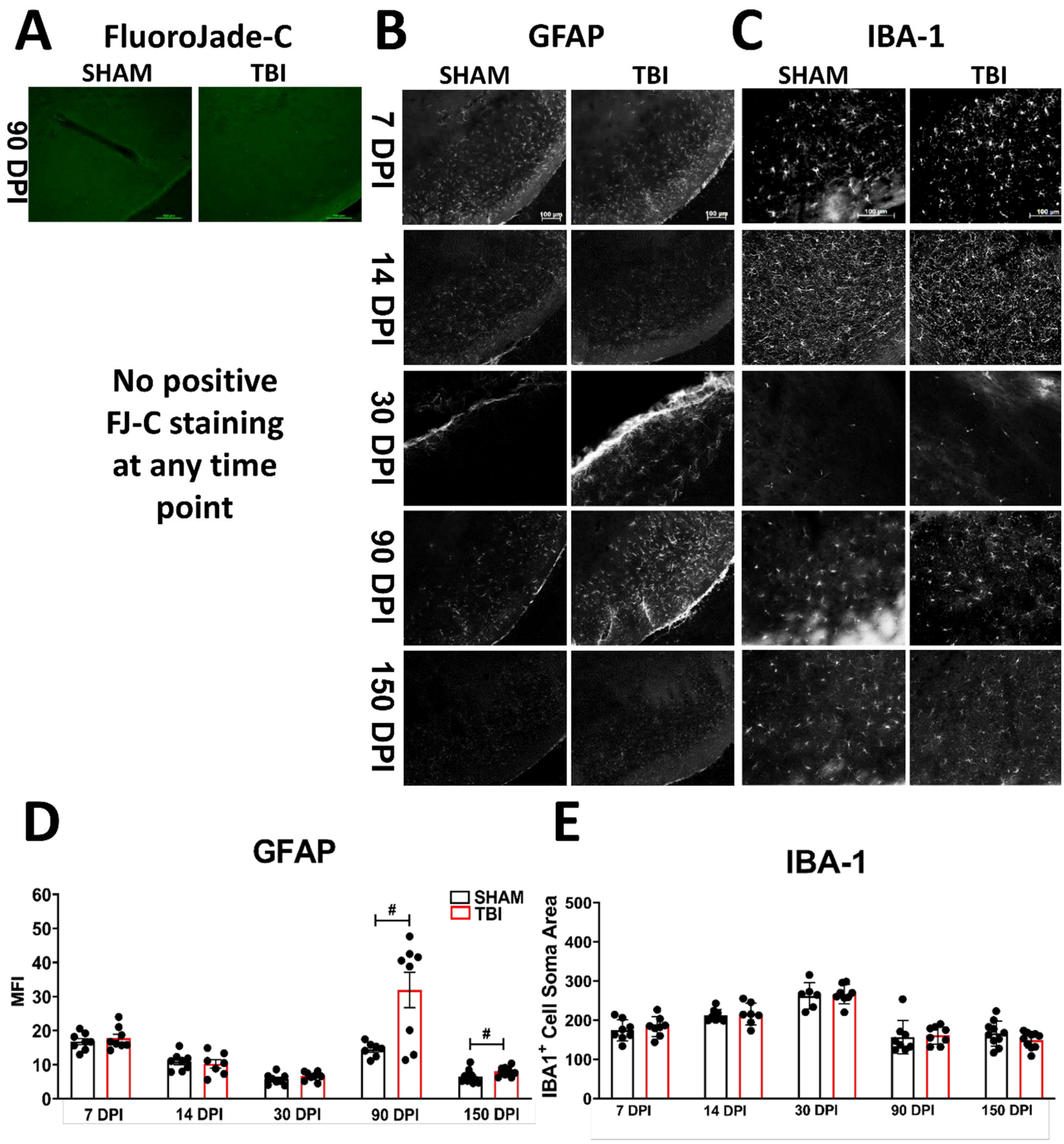

Figure S20. The substantia nigra *pars reticulate* at 90 DPI showed astroglial response to injury. (A) There was no degeneration in this region of the thalamus for which all images looked like the ones depicted here from 90 DPI. (B) Photomicrographs of GFAP at each time point (10x) show no differences save for at 90 DPI (D). (C) Shows 20x images of IBA-1, where there was no immune response to TON in the SNpr at any time (E). Scale bars indicate 100µm. # $p<0.05$

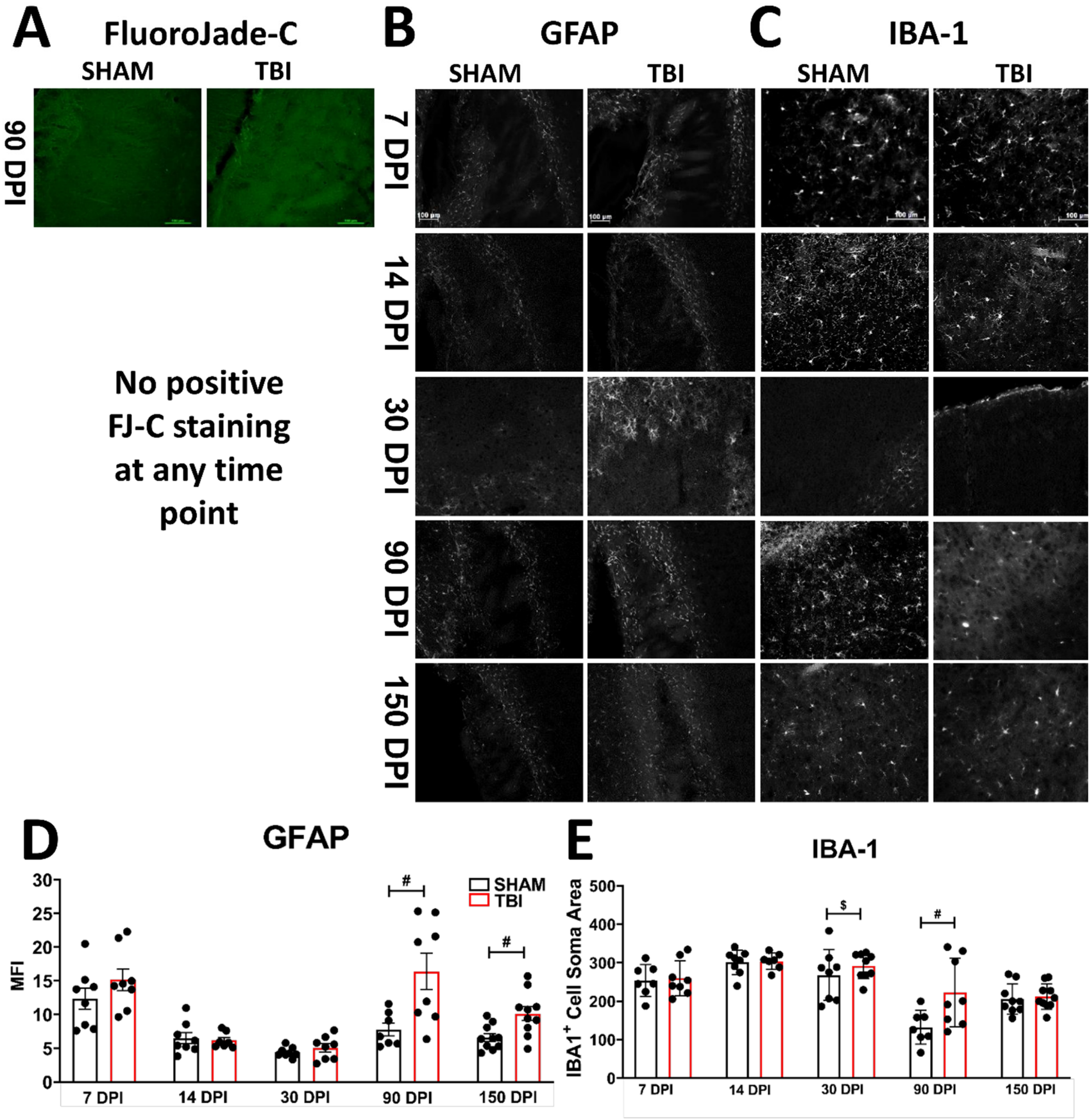

Figure S21. The caudoputamen responds to OT injury staring at 90 DPI until the end of the time course. (A) There was no degeneration in this region of the thalamus for which all images looked like the ones depicted here from 90 DPI. (B) Photomicrographs of GFAP at each time point (10x) show no differences save for at 90 and 150 DPI (D). Interestingly, the astrocyte morphology at 30 DPI, while not significantly more intense, does show similar characteristics to those shown in the visual cortex. (C) Shows 20x images of IBA-1, where there was no immune response to TAI in the CP at any time (E). Scale bars indicate 100μm. # $p<0.05$
